# Bolstered Interfacial Field Chemistry for Deep Fast-Charging Aqueous Zinc Metal Batteries

**DOI:** 10.1007/s40820-026-02314-5

**Published:** 2026-07-29

**Authors:** Minxi Sun, Yining Chen, Congge Lu, Jingkang Ma, Qiuyuan Feng, Shaoxing Li, Tao Zhang, Shuang Zhou, Anqiang Pan

**Affiliations:** 1 School of Materials Science and Engineering, Key Laboratory of Electronic Packaging and Advanced Functional Materials of Hunan Province, Central South University, Changsha, 410083 People’s Republic of China; 2 Xinjiang Key Laboratory of Advanced Metallic Materials Design and Application, Xinjiang Engineering Research Center of Environmental and Functional Materials, School of Materials Science and Engineering, Xinjiang University, Urumqi, 830046 People’s Republic of China

**Keywords:** Aqueous zinc mental batteries, Inner Helmholtz plane regulation, Fast-charging, Depth of discharge, Interfacial adsorption

## Abstract

Deep fast-charging capability has become the core pursuit for practical applications of aqueous zinc metal batteries, yet it is critically impeded by unfavorable interfacial field evolution at the electrode/electrolyte interface under high-current–density conditions. The coexistence of sluggish Zn^2+^ desolvation, competitive H_2_O reduction corrosion, uneven electric field, and concentration field collectively leads to poor rate performance. To tackle these issues, a bolstered interfacial field chemistry strategy was developed via sulfosuccinic acid to precisely regulate the physicochemical and electrochemical properties within the inner Helmholtz plane. The tuned interfacial field comprises a homogeneous distribution of interfacial ions and electric fields, promoting fast desolvation and interfacial electron transfer. Consequently, the modified Zn||Cu asymmetric cells deliver an outstanding average Coulombic efficiency of 99.48% over 1600 cycles at 2 mA cm^−2^ and 1 mAh cm^−2^. Furthermore, the modified Zn||Zn symmetric cells demonstrate exceptional stability under challenging conditions of 5 mA cm^−2^, 2 mAh cm^−2^ (over 1600 h), and 10 mA cm^−2^, 10 mAh cm^−2^ (over 675 h, depth of discharge = 17.08%). Impressively, a substantial cumulative capacity of 3500 mAh cm^−2^ is attained at 10 mAh cm^−2^ and a 56.93% Zn utilization rate. Besides, the enhanced Zn (10 µm) ||I_2_ (10.87 mg cm^−2^) full cell sustains over 1490 cycles at 1 A g^−1^ with 77.13% capacity retention and a harsh N/P ratio of 2.31. Remarkably, the Zn (10 µm) ||I_2_ pouch cell achieves over 680 cycles with an ultralow N/P ratio of 2.04.

**Supplementary Information:**

The online version contains supplementary material available at 10.1007/s40820-026-02314-5.

## Highlights

- A sulfosuccinic acid-enabled interfacial field chemistry strategy is developed to regulate the inner Helmholtz plane, which homogenized ion/electric distribution, thus accelerating Zn^2+^ desolvation and charge transfer under high current density.
- This strategy enables superior reversibility and ultralong stability in Zn||Zn cells, simultaneously meeting the requirements of high current density and deep discharge.
- The optimized Zn (10 µm) ||I_2_ full cells deliver over 1490 cycles (N/P = 2.31) and 680 cycles in pouch cells (N/P = 2.04), respectively.

**Supplementary Information:**

The online version contains supplementary material available at 10.1007/s40820-026-02314-5.

## Graphical abstract

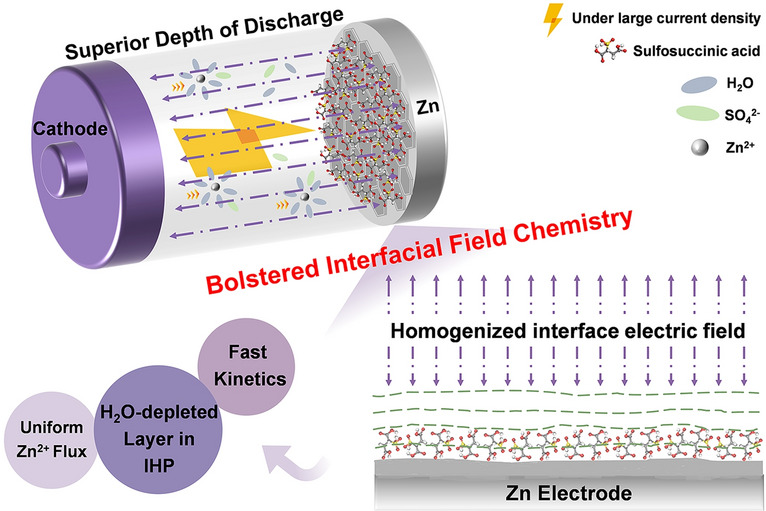

## Introduction

Worldwide climate change and the collective goal of carbon neutrality have spawned the third energy revolution [1], where advanced electrochemical energy storage devices serve as the indispensable cornerstone for accommodating intermittent and unstable renewable power sources [2–4]. Aqueous zinc metal batteries (AZMBs) stand out as one of the leading candidates for next-generation energy storage technologies, attributed to their inherent non-flammability, abundant Zn reserves, and ultrahigh theoretical capacity of Zn metal anodes (820 mAh g^−1^) [5–7]. With the long-standing bottlenecks such as Zn dendrite growth being continuously alleviated through rational electrode/electrolyte engineering, the pursuit of deep fast-charging capability has become the core technological pursuit for practical deployment of AZMBs toward high-power applications. At industrially relevant high current densities (≥ 10 mA cm^−2^), the Zn^2+^ plating/stripping behavior is predominantly governed by interfacial kinetic processes, calling for innovative and targeted strategies to break the kinetic limits of deep fast-charging Zn metal anodes [8].

**Fig. 1:**
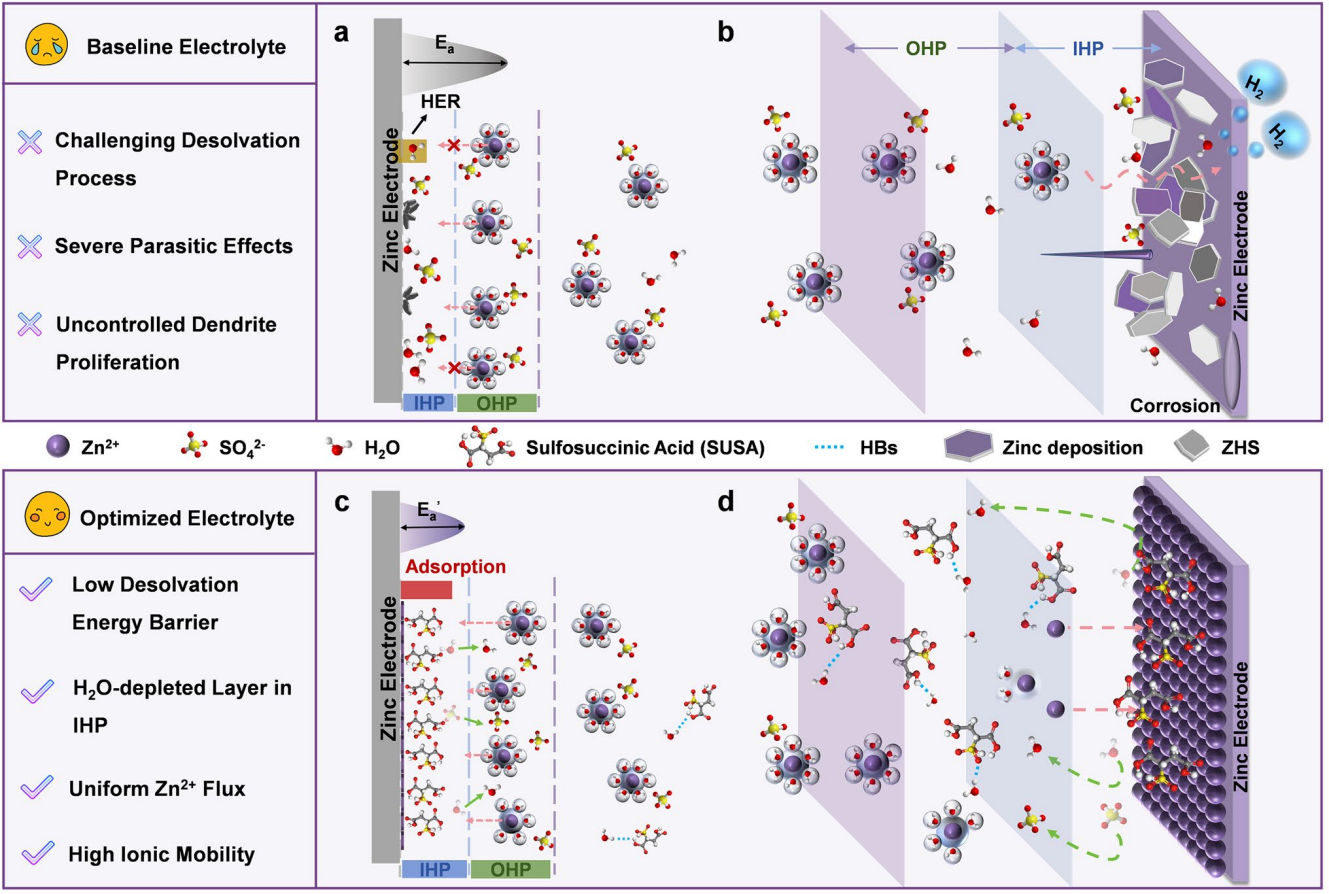
Schematic of Zn deposition process in **a, b** traditional baseline electrolyte of ZnSO_4_ and **c, d** design strategy for modulating Zn^2+^ deposition behavior for highly reversible Zn anodes through SUSA addition

The well-accepted “Space charge” model clarifies that a self-reinforced ion-depleted space charge layer can be rapidly formed within an extremely short Sand’s time [9] under high-current–density conditions. Such detrimental interfacial evolution is closely correlated with the consecutive steps of bulk-phase ion migration, interfacial desolvation, and charge transfer. In conventional aqueous electrolytes, Zn^2+^ is strongly coordinated by six H_2_O molecules within its primary solvation sheath [10], which imposes a formidable energy barrier for interfacial desolvation and subsequent charge transfer (Fig. 1a) [11]. The sluggish desolvation process inevitably gives rise to severe interfacial concentration polarization, resulting in a distortion of the electric field homogeneity at the interface. This inhomogeneity can be dramatically aggravated by surface defects and preferential nucleation sites [12, 13], leading to the coupled deterioration of potential and concentration fields at the electrode/electrolyte interface [14]. Consequently, the uncontrolled co-occurrence of Zn dendrite growth, surface passivation, and hydrogen evolution reaction (HER) severely undermines the structural integrity and interfacial stability of Zn anodes (Fig. 1b), resulting in drastically increased interfacial impedance, aggravated polarization, and the eventual failure of high-rate operation [15–18].

Researchers have made extensive efforts to promote bulk Zn^2+^ migration and accelerate interfacial desolvation kinetics [19–23]. Nevertheless, the comigration and competitive diffusion between Zn^2+^ and proton cations (H^+^) in aqueous electrolytes make it difficult to distinguish or individually regulate the directional transport of Zn^2+^, thereby hindering the precise control of cation migration kinetics. Although remarkable progress has been achieved in facilitating interfacial desolvation and charge transfer of hydrated Zn^2+^ [24–26], the construction of Zn anodes with superior high-rate stability, long-term reversibility, and high depth of discharge (DOD) remains a critical challenge, especially under practical harsh conditions including high current densities and elevated temperatures [27, 28]. It is widely acknowledged that the inner Helmholtz plane (IHP), composed of adsorbed solvent molecules and anions, acts as the critical reaction arena for interfacial desolvation and charge transfer. Therefore, in-depth understanding and targeted manipulation of the physicochemical and electrochemical properties of the electrode/electrolyte interfacial field will provide a distinctive and effective paradigm for developing highly reversible and deep fast-charging Zn metal anodes toward high-power aqueous energy storage.

Herein, we propose a bolstered interfacial field chemistry strategy to rationally engineer the electrode/electrolyte interfacial microenvironment, especially the IHP, for realizing highly stable and deep fast-charging Zn metal anodes. Without compromising the “migration-viscosity” trade-off, an eco-friendly and nearly ultra-trace (0.36%) additive (sulfosuccinic acid, denoted as SUSA) was incorporated into the conventional 2 M ZnSO_4_ (denoted as ZSO) electrolyte. Driven by its intrinsic Zn affinity, SUSA adsorbs in an enhanced mode within the IHP, expelling H_2_O and thereby inhibiting HER corrosion and side reactions. The optimized interfacial field effectively homogenizes the ion flux and electric field distribution, thereby accelerating Zn^2+^ desolvation and charge transfer kinetics. Thus, this strategy alleviates the space charge layer effect at large current densities, inducing uniform Zn deposition and the formation of an ordered (002) texture (Fig. 1c, d). Additionally, SUSA also modifies the aqueous hydrogen bond (HB) network, enhancing Zn anode cyclability at low temperature of − 20 °C as well as at high temperature of 60 °C (over 300 h for long-cycling operation). Moreover, SUSA imparts high reversibility, achieving an average Coulombic efficiency (CE) of 99.48% over 1600 cycles at 2 mA cm^−2^ and 1 mAh cm^−2^. These effects collectively yield high stability in modified Zn||Zn symmetric cells at 5 mA cm^−2^, 2 mAh cm^−2^ (over 1600 h) and 10 mA cm^−2^, 10 mAh cm^−2^ (over 675 h, DOD = 17.08%). Impressively, the symmetric cells using lean Zn (30 μm) exhibited a substantial cumulative capacity of 3500 mAh cm^−2^ with a high area capacity of 10 mAh cm^−2^ and a high Zn utilization rate of 56.93%. The Zn (10 µm) ||I_2_ (10.87 mg cm^−2^) coin full cell cycled exhibited cycling stability over 1490 cycles at 1 A g^−1^ with 77.13% capacity retention under an N/P ratio of 2.31. Notably, a pouch-based full cell configuration (Zn: 10 µm, I_2_: 12.54 mg cm^−2^) achieved over 680 cycles with 70.17% retention at an ultralow N/P ratio of 2.04. This minimal-effective electrolyte additive initiative effectively elevates Zn anode stability through the theory of dynamic bimodal synergistic adsorption framework. The strategy reveals the critical role of interfacial field chemistry in high-rate Zn^2+^ deposition behavior and drives the progression of high-capacity, sustained-cycle AZMBs at economical costs, delivering a viable blueprint for their efficient and practical utilization.

## Experimental Section

### Electrolyte Preparation

The ZSO electrolyte (2 M ZnSO_4_) was prepared by dissolving 5.7512 g ZnSO_4_ (≥ 99.5%, Sinopharm Chemical Reagent Co., Ltd.) in deionized water (DI water) and diluting to a final volume of 10 mL after complete dissolution. The ZSO/SUSA series electrolytes were prepared by dissolving 5.7512 g of ZnSO_4_ and 0.0283, 0.0566, 0.0849, and 0.1132 g (corresponding to SUSA solutions with concentrations of 0.01, 0.02, 0.03, and 0.04 mol L^−1^, respectively) of SUSA (sulfosuccinic acid, 70 wt% in H_2_O, Macklin), respectively, in DI water until completely dissolved, and then diluting to a final volume of 10 mL to obtain the solution named ZSO/SUSA-0.01, ZSO/SUSA-0.02, ZSO/SUSA-0.03, and ZSO/SUSA-0.04.

### Preparation of I_2_ Cathode

Mix tetrachloroethyl iodide and iodine in a 1:1 ratio, grind thoroughly, and react at 120 °C for 24 h. After grinding the resultant materials completely, homogeneously mix the above material with activated carbon (AC), Kochin black (KB, EC600JD), and polytetrafluoroethylene (60%, PTFE) in a mass ratio of 8:8:1:1 in a suitable amount of 1-propanol until a moldable consistency is achieved. The formed composite is then punched into 10-mm-diameter disks or square pieces of specified dimensions for the fabrication of coin cells and pouch cells. Finally, the materials were dried in a 50 °C forced-air drying oven for 12 h, affording the I_2_ cathode sheets.

## Results and Discussion

**Fig. 2:**
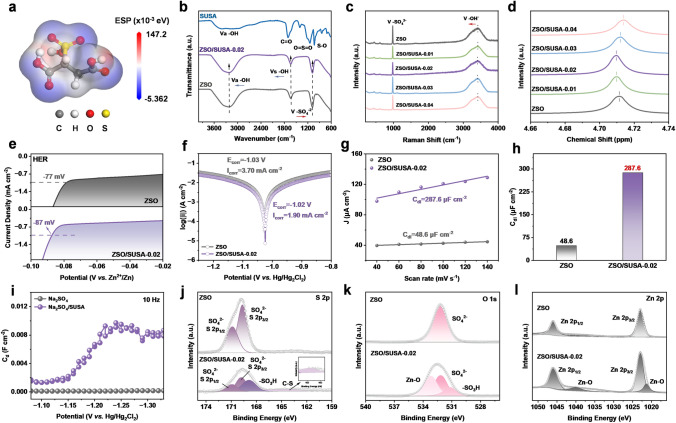
Effect of SUSA on electrolyte properties. **a** MESP distribution of SUSA molecule. **b** FTIR spectral analysis of SUSA aqueous solution, ZSO/SUSA-0.02 and ZSO electrolytes. **c** Raman spectra for ZSO, ZSO/SUSA-0.01, ZSO/SUSA-0.02, ZSO/SUSA-0.03 and ZSO/SUSA-0.04 electrolytes. **d** ^1^H NMR spectral analysis for ZSO, ZSO/SUSA-0.01, ZSO/SUSA-0.02, ZSO/SUSA-0.03, ZSO/SUSA-0.04. **e** LSV profiles of ZSO and ZSO/SUSA-0.02. **f** Zn electrode’s Tafel curves in ZSO with/without SUSA. **g** Linear fitting for EDLC calculation in various electrolytes and corresponding **h** comparative numerical diagram. **i** Changes in EDL capacitance of Na_2_SO_4_ and Na_2_SO_4_/SUSA electrolytes measured in a three-electrode system. XPS analysis of Zn anode electroplated for 5 min at 1 mA cm^−2^: **j** S 2*p*, **k** O 1* s*, **l** Zn 2*p*

As enumerated in Fig. 2a, the oxygen atoms in the SUSA molecules exhibit relatively low electrostatic potential (ESP) due to their internal lone pair electrons, demonstrating the affinity of SUSA for Zn. This indicates that it can adhere to the Zn surface via zincophilic carboxyl group adsorption [29, 30]. A series of electrolyte solutions with different concentration gradients were formulated (denoted as ZSO/SUSA-0.01, ZSO/SUSA-0.02, ZSO/SUSA-0.03, ZSO/SUSA-0.04). To investigate its role in the bulk electrolyte at the microscopic molecular level, a variety of spectroscopic studies were conducted using electrolytes with SUSA. As shown by Fourier-transform infrared (FTIR) spectroscopy in Figs. 2b and S1, the blueshift of the H–O stretching vibration bond (Va-OH, from 3216.56 cm^−1^ of ZSO to 3223.79 cm^−1^ of ZSO/SUSA-0.02) within the approximate range of 2800–3800 cm^−1^ indicates the disruption of the primordial HB network among H_2_O. Meanwhile, the –OH bending mode (Vs-OH, within approximately 1550–1760 cm^−1^) also shows a faint blueshift (from 1636.35 cm^−1^ of ZSO to 1636.50 cm^−1^ of ZSO/SUSA-0.02), suggesting the attenuation of aqueous chemical reactivity [31]. Notably, this blueshift trend is consistently observed across electrolytes with different SUSA concentrations, confirming the general capability of the additive in modulating the HB network. And the outcomes of Raman are consistent with FTIR (Fig. 2c). A redshift of v–OH indicates the HBs between H_2_O–H_2_O have been weakened. Consequently, due to the inherent abundance of HB donors and acceptors, SUSA molecules tend to interact with H_2_O to form HBs, thereby disrupting the original HB-network structure in the electrolyte system. This conclusion is further evidenced by the nuclear magnetic resonance (NMR) in Fig. 2d. The ^1^H peak of ZSO is positioned at 4.711 ppm, and the peak is shifted to 4.710 ppm in ZSO/SUSA-0.02 electrolyte. This shielding effect is attributed to the interaction of SUSA-H_2_O, increasing the electron cloud density of the electrolyte and weakening the polarization effect between Zn^2+^ and H_2_O. The reduction in H_2_O activity consequently leads to an improvement in ionic transference [32]. When increasing additive concentration, the strong polarity of SUSA leads to increased formation of SUSA with H_2_O (SUSA-H_2_O-SUSA), causing the strengthening of the HB network within the system [33]. Due to the competition among H_2_O molecules, the O–H electron cloud is pulled apart, resulting in the blueshift of ^1^H peaks of ZSO/SUSA-0.03 and ZSO/SUSA-0.04 electrolytes. Owing to the limited chemical reactivity of H_2_O and the adsorption of SUSA on the electrode, the HER of ZSO/SUSA-0.02 electrolyte is inhibited (Figs. 2e and S2). In summary, 0.02 M SUSA is an ideal additive dosage for ZSO electrolyte, and we mainly used ZSO/SUSA-0.02 electrolyte for our follow-up research.

Tafel test was taken to prove the corrosion resistance of SUSA (Fig. 2f). The improved corrosion potential (from − 1.03 V of ZSO to − 1.02 V of ZSO/SUSA-0.02) and significantly reduced corrosion current (from 3.70 mA cm^−2^ of ZSO to 1.90 mA cm^−2^ of ZSO/SUSA-0.02) underscore the remarkable contribution of SUSA adsorption to the suppressive effect on Zn self-corrosion. The static immersion test further confirmed the role of SUSA in enhancing the thermodynamic stability of Zn anodes. As shown in Fig. S3, after 7 days of static immersion at room temperature, the Zn foil soaked in ZSO electrolyte suffers from severe surface corrosion, accompanied by obvious corrosion pits. The corresponding scanning electron microscope (SEM) and X-ray diffraction (XRD) results confirm the generation of massive passivation products. On the contrary, the Zn foil immersed in ZSO/SUSA-0.02 electrolyte maintains a flat and intact surface with negligible corrosion traces.

To gain more insight, the electric double-layer capacitance (EDLC, C_EDL_) was determined to elucidate the adsorption behavior of SUSA (Figs. 2g and S4). The value of C_EDL_ of ZSO/SUSA-0.02 electrolyte demonstrates a nearly six-fold enhancement compared with that of the ZSO electrolyte (Fig. 2h). The outcome suggests that SUSA, as a polar additive, forms a tight compactly bound electrostatic adsorption layer on the electrode surface, creating an increased quantity of Zn^2+^ nucleation sites [34]. This enables Zn^2+^ to form uniform and dense deposits at the Zn/electrolyte interface. Based on the traditional double-layer capacitance theory, to further investigate the adsorption-regulating capability of SUSA and minimize the interference from Faraday current, 1 M Na_2_SO_4_ was chosen to replace 2 M ZnSO_4_ as the base solvent (denoted as Na_2_SO_4_ and Na_2_SO_4_/SUSA), and the EDL test was conducted by a three-electrode system with the novel electrolyte systems (Fig. S5). Applying the same amplitude at dynamic potentials, the capacitance value of Na_2_SO_4_/SUSA has a sudden surge compared to Na_2_SO_4_ (Fig. 2i). This points to the adsorption of SUSA in the IHP [35], completing the structure of the electric double layer (EDL) and directing a H_2_O-depleted layer. In essence, the trace introduction of SUSA leads to the reconfiguration of molecular adsorption pattern at the interface and the inhibition of the subsequent adverse reactions.

The adsorption type of SUSA was further investigated by X-ray photoelectron spectroscope (XPS) analysis of the Zn electrode electroplated for 5 min at 1 mA cm^−2^ (Fig. S6). From S 2*p* spectrum (Fig. 2j), the appearance of -SO_3_H and C-S peaks confirms the static adsorption of SUSA molecules. Notably, the signal of –SO_4_^2−^ on the Zn surface within ZSO/SUSA-0.02 electrolyte indicates significant weakening compared to that within ZSO electrolyte, suggesting that the introduction of SUSA can effectively adsorb and therefore remove SO_4_^2−^ from the IHP layer [36]. This trend is also clearly observed in the O 1*s* spectrum (Fig. 2k). And the Zn–O peak serves as evidence for charge transfer between SUSA and electrode, substantiating its electrochemical adsorption behavior. The carboxyl group in the molecule can be deprotonated to form strong coordination with the Zn surface and even quasi-covalent bonds, achieving bolstered stable electrochemical adsorption [37]. As illustrated in Fig. 2l, after introduction of SUSA, the 2*p*_1/2_ (1045.75 eV) and 2*p*_3/2_ (1022.72 eV) peaks of Zn enhance, indicating that Zn^2+^ rapidly transfers to the electrode/electrolyte interface to form deposits [38]. This synergistic combination of robust electrochemical adsorption and physical adsorption homogenizes the interface electric field and enhances ion migration, thereby underpinning the enhanced electrochemical stability.

**Fig. 3:**
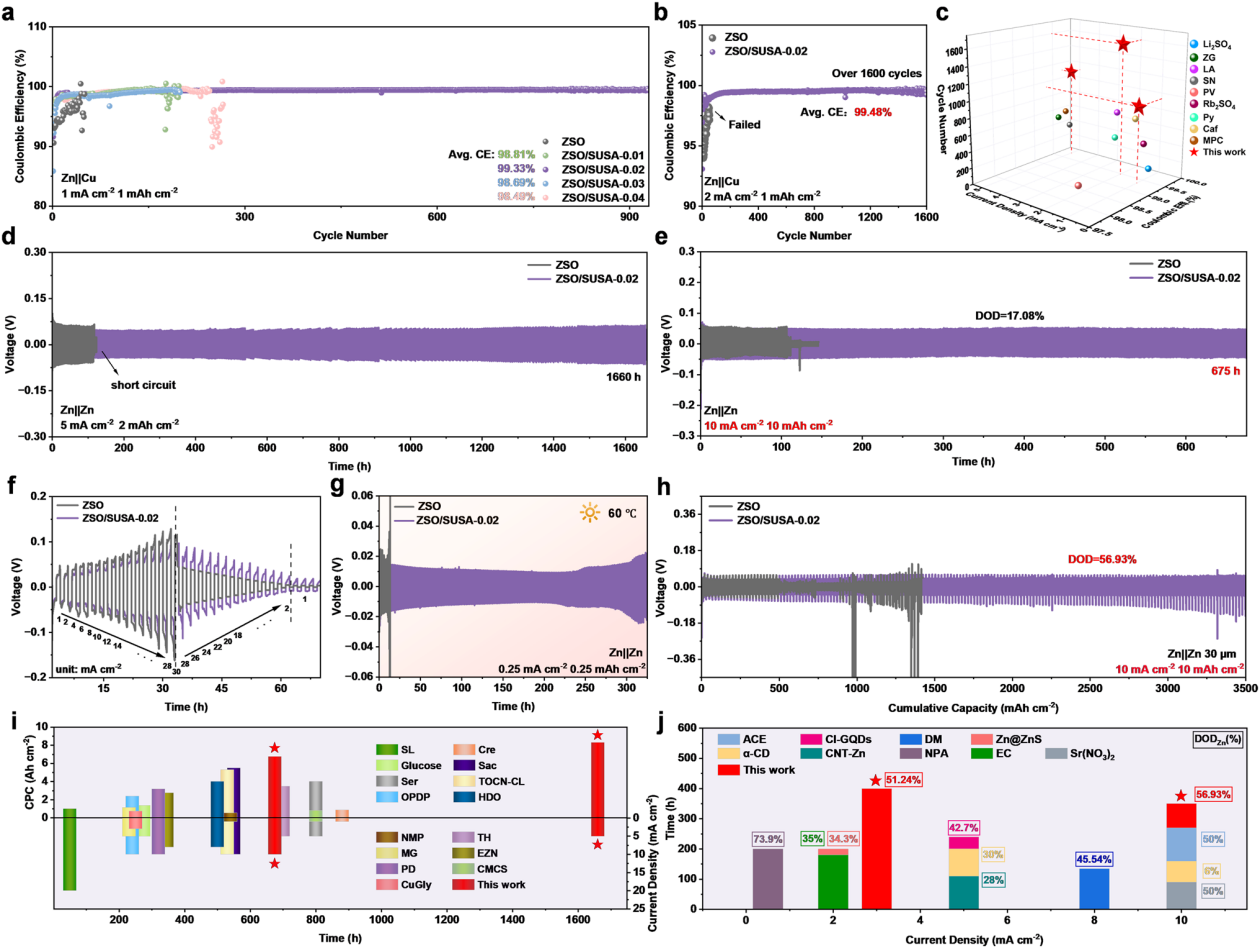
Zn||Cu and Zn||Zn cells performance within various electrolytes. CE of Zn||Cu half cells in ZSO with/without SUSA electrolytes cycled under **a** 1 mA cm^−2^ and 1 mAh cm^−2^,** b** 2 mA cm^−2^ and 1 mAh cm^−2^. **c** Performance of this work (cycle number, current density, CE) benchmarked against prior literature. Electrochemical performance characterization of Zn||Zn symmetric cells in ZSO with/without SUSA electrolytes cycled under **d** 5 mA cm^−2^ and 2 mAh cm^−2^, **e** 10 mA cm^−2^ and 10 mAh cm^−2^ (DOD≈17.08%), **f** various rate, **g** 0.25 mA cm^−2^ and 0.25 mAh cm^−2^ at high temperature of 60 °C, **h** 10 mA cm^−2^ and 10 mAh cm^−2^ (DOD≈56.93%). **i, j** Comparison of the cycling performance of Zn||Zn symmetric cells in this work with other documented assembled batteries in various optimized electrolytes

For the purpose of in-depth exploration of SUSA’s role in boosting the stability of electrode, Zn||Cu asymmetric cells were assembled for testing at different current densities. At the trickle condition of 1 mA cm^−2^, 1 mAh cm^−2^, the cells incorporating ZSO/SUSA-0.02 electrolyte complete over 900 cycles with a pronounced average CE of 99.33%, substantially superior to counterparts with ZSO alone and other SUSA-concentration electrolytes (Fig. 3a). Elevating to 2 mA cm^−2^ leads to evident CE variations in Zn||Cu cells within ZSO electrolyte, resulting in failure after just 53 cycles due to internal intense side effects. In stark contrast, the ZSO/SUSA-0.02 electrolyte allows cells to sustain over 1600 cycles at an exceptional average CE of 99.48% and shows highly overlapping voltage curves versus ZSO electrolyte (Figs. 3b and S7), indicating that it has markedly enhanced the reversibility of the Zn anode. And when operating under 5 mA cm^−2^ (1 mAh cm^−2^), the excellent reversibility of Zn anode enables the system’s CE to exhibit the same trend (Fig. S8). The ZSO/SUSA-0.02 electrolyte significantly boosts the anode plating/stripping reversibility, outperforming most documented results and delivering persuasive experimental evidence for the SUSA additive’s outstanding efficacy (Fig. 3c and Table S1).

Thus, Zn||Zn symmetric cells tested under multiple current densities were fabricated to evaluate their long-cycle performance. The assembled ZSO/SUSA-0.02-based cells demonstrate excellent cycling stability under low-current conditions of 1 mA cm^−2^, maintaining cycling performance for over 1500 h (Fig. S9). As displayed in Fig. 3d, at 5 mA cm^−2^ and 2 mAh cm^−2^, the cells using ZSO electrolyte experience abrupt failure after no more than 120 h, whereas the ZSO/SUSA-0.02 electrolyte enables endurance past 1660 h. Notably, even at a large current density at 10 mA cm^−2^ and a substantial capacity of 10 mAh cm^−2^, the Zn||Zn cells employing ZSO/SUSA-0.02 electrolyte support astonishing cycling over 675 h, significantly outperforming those in the baseline electrolyte and in the electrolytes with other SUSA concentrations (Figs. 3e and S10). More importantly, under this condition, an astonishing cumulative plating capacity (CPC) of up to 6.75 Ah cm^−2^ was achieved at 17.08% depth of discharge (DOD). Following the encouraging outcomes, the current density was heightened to 20 mA cm^−2^ (10 mAh cm^−2^). The ZSO/SUSA-0.02-based cells deliver stable performance exceeding 340 h with steady polarization (Fig. S11). Further, rate performance tests were performed to assess the tolerance of the modified electrolyte to high current densities. Upon ramping the current density from 1 to 30 mA cm^−2^, the ZSO/SUSA-0.02-based cells maintain stable voltage curves and minimal hysteresis compared to ZSO-based cells, with seamless return to 1 mA cm^−2^ (Figs. 3f and S12). The wide current-capacity adaptability of this enhanced adsorption interface was confirmed.

Notably, SUSA also significantly enhances the system’s operational temperature range. Rate performance tests under varying temperatures reveal that ZSO-based cells fail instantly when cooled below − 20 °C. In stark contrast, the cells within ZSO/SUSA-0.02 electrolyte maintain stable from − 20 to 60 °C, demonstrating reliable all-climate functionality throughout a seamless transition from sub-zero conditions to high temperatures (Fig. S13). The differential scanning calorimetry (DSC) test shows that the freezing point of ZSO/SUSA-0.02 electrolyte is lower than that of ZSO electrolyte by 17.2 °C (Fig. S14), indicating a great contribution of SUSA to expand the service temperature range. Even at a high temperature of 60 °C, the ZSO/SUSA-0.02-based cells could cycle stably beyond 300 h under 0.25 mA cm^−2^ (0.25 mAh cm^−2^), which significantly prolongs cycling life and greatly broadens the working temperature of the system (Fig. 3g). Corresponding SEM images reveal that the cells cycled in the ZSO electrolyte experienced a substantial amount of by-products observable internally and an uneven, rugged cross section (Fig. S15a). In contrast, the cells cycled in the ZSO/SUSA-0.02 electrolyte maintained relatively flat and uniform surfaces and cross sections even after 50 and 100 cycles (Fig. S15b, c). This demonstrates that the bolstered interfacial adsorption of SUSA, combined with its ability to modulate the HB network in the bulk electrolyte, confers outstanding high-temperature cycling stability to the Zn anode.

For thorough practical assessment via DOD testing, symmetric Zn||Zn cells incorporating thin Zn foils were fabricated. Impressively, as illustrated in Figs. 3h and S16, the symmetric cell using lean Zn (30 μm) exhibited a substantial CPC of 3.5 Ah cm^−2^ under the strict condition of 10 mA cm^−2^, 10 mAh cm^−2^, and a high Zn utilization rate of 56.93%. And at 3 mA cm^−2^ and 3 mAh cm^−2^ (DOD = 51.24%), additive-free cells experience rapid overpotential escalation, while the cells in the ZSO/SUSA-0.02-enhanced electrolyte endure over 380 h (Fig. S17). A comparable pattern is evident across 85.40% DOD levels (Fig. S18), suggesting that SUSA additives facilitate substantial Zn anode lifespan prolongation, even under stringent operating conditions. As evidenced by the prior data, the SUSA additives effectively enhance Zn anode longevity under elevated current, large capacity, and harsh conditions. The assembled ZSO/SUSA-0.02-based cells present extra CPC (6.75 Ah cm^−2^, 8.3 Ah cm^−2^) and endurance under high-DOD conditions (51.24%, 56.93%), surpassing the majority of existing reports (Fig. 3i, j, Tables S2 and S3). This enhanced interface implemented by SUSA shows strong potential to meet commercial demands for deep fast-charging.

**Fig. 4:**
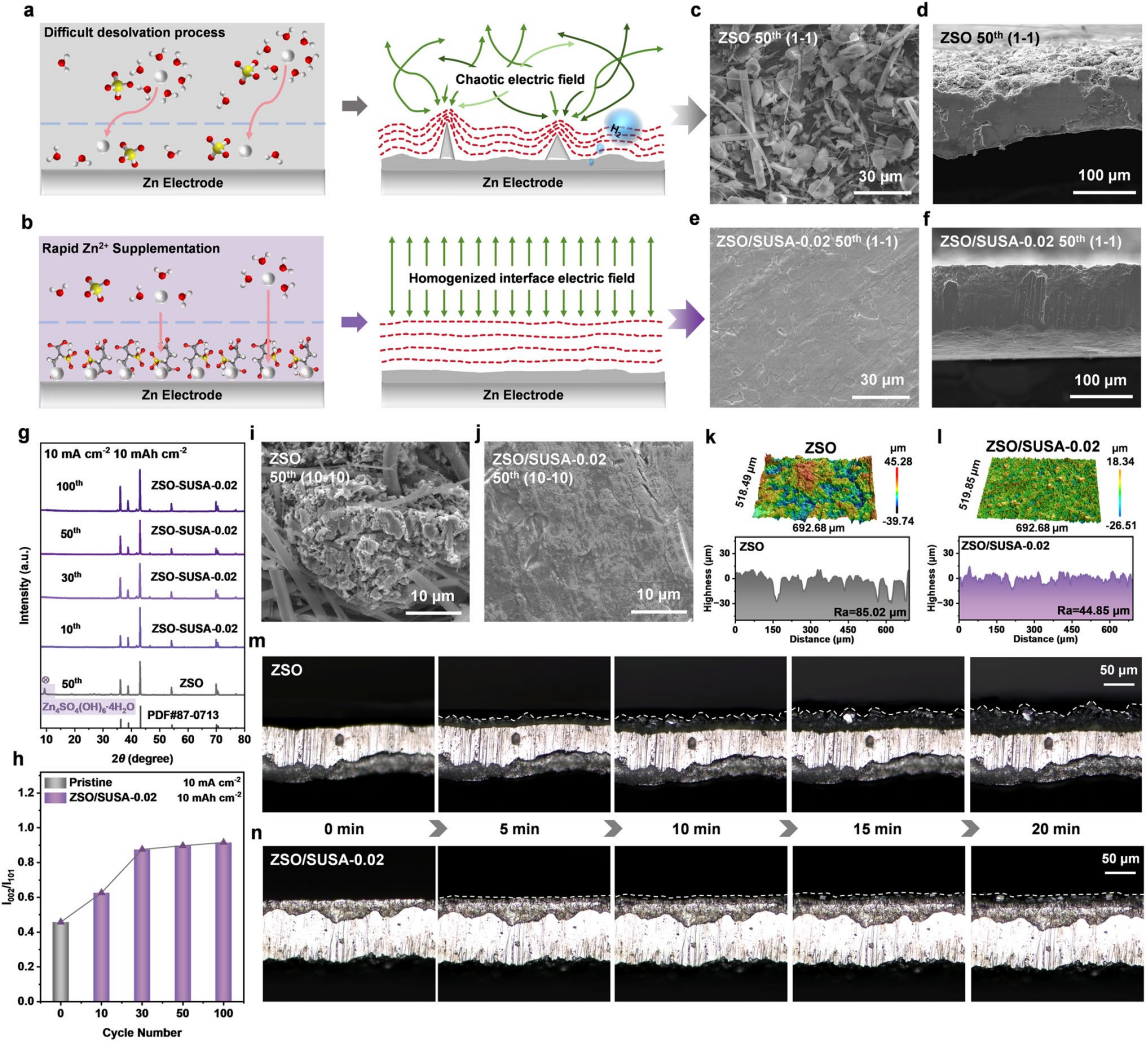
Morphology and surface structural alterations of Zn electrode within ZSO and ZSO/SUSA-0.02 electrolytes. Schematic diagram of the IHP layer at the interface and the corresponding electric field distribution in **a** ZSO electrolyte and **b** ZSO/SUSA-0.02 electrolyte. SEM images of the frontal and cross-sectional views of the Zn electrode after 50 cycles at 1 mA cm^−2^ and 1 mAh cm^−2^ in **c, d** ZSO electrolyte and **e, f** ZSO/SUSA-0.02 electrolyte. **g** XRD patterns of Zn anode after designated cycle numbers under 10 mA cm^−2^ and 10 mAh cm^−2^ in various electrolytes and corresponding **h** bar chart of I_(002)_/I_(101)_ ratio of cycled Zn in ZSO/SUSA-0.02 electrolyte. Post-50-cycle SEM images at 10 mA cm^−2^ and 10 mAh cm^−2^ within **i** ZSO electrolyte, **j** ZSO/SUSA-0.02 electrolyte and corresponding LSCM images of Zn anodes in **k** ZSO electrolyte, **l** ZSO/SUSA-0.02 electrolyte. In situ optical microscopy of Zn anodes for 20-min deposition at 5 mA cm^−2^ in **m** ZSO electrolyte and **n** ZSO/SUSA-0.02 electrolyte

We therefore focused on the morphological and compositional changes of the electrode to uncover the underlying causes for the enhanced cycling stability. Through deprotonation and coordination of the carboxyl group with Zn atoms, SUSA exhibits enhanced adsorption at the Zn anode interface, developing a water-poor IHP layer with a homogenized local electric field. Therefore, the Zn^2+^ flux is induced by the uniform electric field, leading to compact and uniform deposition. This is in contrast to the “tip effect” of Zn^2+^ deposition in common ZSO electrolyte where the IHP is occupied by water molecules (Fig. 4a, b). Consequently, SEM imaging discloses abundant corrosion residues on the surface, which were characterized by a loose and heterogeneous morphology with sections showing collapse, correlating with diminished electrochemical performance in Zn||Zn cells (Fig. 4c, d). Conversely, the Zn anode within ZSO/SUSA-0.02 electrolyte exhibits a compact and smooth appearance (Fig. 4e, f).

At a high current density and deep capacity (10 mA cm^−2^, 10 mAh cm^−2^), XRD analysis reveals the presence of distinct by-products of Zn_4_SO_4_(OH)_6_·4H_2_O (ZHS, PDF#44-0673) in the ZSO-based batteries after 50 cycles. In contrast, by-product formation remains minimal in the ZSO/SUSA-0.02 electrolyte, alongside an upward trajectory in the I_(002)_/I_(101)_ ratio as cycles accumulate (Figs. 4g, h and S19). Consequently, the introduction of SUSA enables the system to adapt to high-current and high-capacity conditions through the excellent Zn^2+^ reaction kinetics, while also inducing Zn^2+^ deposition along the (002) plane and leading to compact Zn deposition. By contrast, Zn anodes in the basic electrolyte undergo severe corrosion, impairing their cycling stability, which is also evident from SEM images of Zn anodes cycled 50 times at 10 mA cm^−2^ and 10 mAh cm^−2^ (Fig. 4i, j). Even after 250 cycles, the surface morphology of the Zn anode remains smooth under the regulation of SUSA as presented in Fig. S20. Laser scanning confocal microscopy (LSCM) results further clarify the differences between them. The surface roughness (Ra) declined from 85.02 μm in the ZSO electrolyte to 44.85 μm in the ZSO/SUSA-0.02 electrolyte (Figs. 4k, l and S21). In situ optical microscopy systems monitored direct evidence of SUSA regulating Zn^2+^ deposition behavior, and following a 20-min deposition at 5 mA cm^−2^, the ZSO-based anode displayed significant uneven deposition, while the electrode in the ZSO/SUSA-0.02 electrolyte remained uniformly consistent throughout (Fig. 4m, n).

Beyond that, the morphology of Zn deposited on Cu cathodes was examined at varying current densities. When under the current density of 2 mA cm^−2^, the optical photograph shows negligible Zn deposition on Cu foils after cycling in the ZSO electrolyte, suggesting difficult Zn^2+^ desolvation. Moreover, it can be observed from corresponding SEM images that the deposited particles are relatively large in size (Fig. S22a). However, after cycling in the ZSO/SUSA-0.02 electrolyte, the Cu surface formed smaller and denser deposit particles (Fig. S22b). As displayed in Fig. S23, the Zn deposit within ZSO electrolyte evolves into an uneven surface with irregular blocks as current density increases. Markedly different, the addition of SUSA enables a dense and homogeneous structure of the Zn^2+^ plating. This improvement stems from the adsorption effect of SUSA, effectively stabilizing the interface for even and dendrite-free deposition. To further characterize the crystallographic orientation of the deposits, the same deposition experiments were conducted on Ti substrates. As shown in Fig. S24a, Zn deposited in the ZSO electrolyte exhibits a disordered, non-uniform, and flocculent morphology. In sharp contrast, the deposition in the ZSO/SUSA-0.02 electrolyte yields a dense, smooth surface with a well-developed layered stacking characteristic of metallic Zn (Fig. S24b). Corresponding XRD analysis reveals that the Zn (002) diffraction peak of Zn deposited in the ZSO/SUSA-0.02 electrolyte is significantly more intense than the peak of Zn (101) (Fig. S25). These observations demonstrate that the SUSA additive effectively homogenizes the interfacial electric field, thereby inducing uniform and stable Zn (002) deposition.

**Fig. 5:**
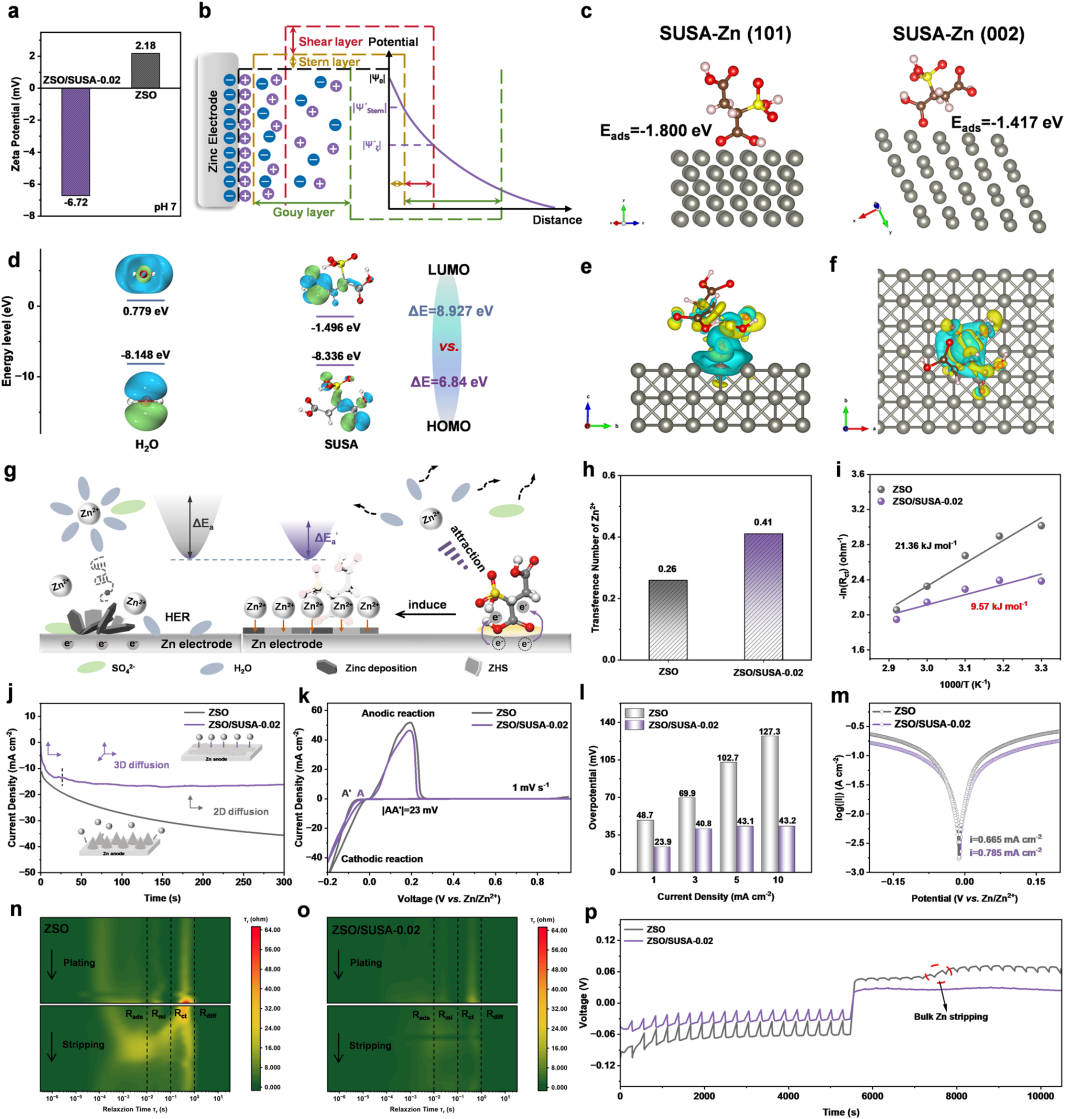
Adsorption behavior and superior kinetics of ZSO/SUSA-0.02 electrolyte. **a** Zeta potential of Zn powder collected from the Zn foil surface after electrodeposition at 1 mA cm^−2^ in ZSO and ZSO/SUSA-0.02 electrolytes. **b** Schematic of EDL in ZSO/SUSA-0.02 electrolyte. **c** Adsorption energy of SUSA on the Zn (101) and Zn (002) facets. **d** LUMO–HOMO energy calculation of H_2_O and SUSA. The differential charge density plots of SUSA on Zn (002) facet from** e** front aspect and **f** top aspect. **g** Schematic diagram of the desolvation and deposition process of Zn^2+^ in varying electrolytes. **h** Transference number of Zn^2+^ in ZSO electrolytes with/without SUSA. **i** Arrhenius curves of activation energy within various electrolytes. **j** CA graphs in ZSO and ZSO/SUSA-0.02 electrolytes.** k** CV curves of Zn||Cu half cells in ZSO and ZSO/SUSA-0.02 electrolytes. **l** Nucleation overpotential under different current densities in ZSO electrolytes with/without SUSA. **m** Corresponding current curves of Zn||Zn symmetric cells within ZSO and ZSO/SUSA-0.02 electrolytes. DRT obtained through in situ EIS within **n** ZSO, **o** ZSO/SUSA-0.02, and **p** corresponding voltage–time curves

Therefore, the synergistic interplay between SUSA adsorption and interfacial ion kinetics merits fundamental and in-depth investigation. To manifest the effects of SUSA on charge distribution, Zn powder collected from the Zn foil surface after electrodeposition at 1 mA cm^−2^ was ultrasonically dispersed to measure the Zeta potential of Zn deposition. From Fig. 5a, the Zn deposited in the ZSO electrolyte displays a positive value, whereas this value decreases to − 6.72 mV following the addition of SUSA. As the diagram illustrates in Fig. 5b, SUSA adsorption triggers a substantial buildup of negative charge on the Zn surface. The oxygen atoms in the carboxyl group and the sulfonyl group, characterized by their high electron cloud density, participate in the reformation of the EDL, thereby reducing the Zeta potential [39]. This negative value of Zn surface in case of ZSO/SUSA-0.02 electrolyte enhances the interaction force between the electrode and Zn^2+^, effectively excluding SO_4_^2−^ and H_2_O [40]. A high absolute Zeta potential promotes uniform nucleation, thereby promoting uniform deposition, which is evident in the SEM images.

The adsorption energies of H_2_O, SO_4_^2−^, and SUSA for Zn (101) and Zn (002) were quantified by density functional theory (DFT) calculations (Figs. 5c, S26, and S27). Notably, SUSA exhibits a more favorable adsorption energy on the Zn (101) surface than on the Zn (002) surface. This preferential adsorption on Zn (101) leads to the greater exposure of the Zn (002) plane, which in turn promotes uniform zinc deposition [41]. The SUSA-adsorbed Zn (101) energy (− 1.800 eV) shows superior strength compared to H_2_O (− 0.484 eV), and likewise, its adsorption on the Zn (002) plane (− 1.417 eV) is also stronger than that of H_2_O (− 0.350 eV). These results substantiate the preferential adsorption of SUSA on both Zn surfaces, which effectively displaces competing H_2_O molecules. As depicted in Fig. 5d, the adsorption of SUSA tightens the lowest unoccupied molecular orbital (LUMO)-highest occupied molecular orbital (HOMO) gap (from 8.927 to 6.84 eV), and the SUSA demonstrates a lower LUMO level (1.496 eV) in contrast to H_2_O (0.779 eV). The strong electron-withdrawing groups (sulfonic acid and carboxyl groups) carried by SUSA reduce its LUMO orbitals and enhance the delocalized electron capacity of the molecule at the interface. Therefore, SUSA can obtain electrons from the Zn electrode [42] and sustain a homogenized local electric field at the interface between the molecule and the anode, facilitating the rapid and homogeneous deposition behavior of Zn^2+^. The yellow part in differential charge density plots also represents the interfacial electron redistribution indicating the bolstered adsorption (both physical and chemical adsorption) between SUSA and Zn anode (Figs. 5e, f, S28 and S29) [43].

Figure 5g summarizes the role of SUSA molecules at the Zn anode/electrolyte interface. In the conventional ZSO electrolyte, sluggish desolvation and heterogeneous deposition of Zn^2+^ initiate a cascade of detrimental events at the interface, including parasitic side reactions with H_2_O and SO_4_^2−^. This process ultimately accelerates rampant Zn dendrite proliferation and acute corrosion at the anode surface. The situation demonstrates a marked improvement in the ZSO/SUSA-0.02 electrolyte. SUSA molecules preferentially adsorb onto the Zn surface through their intrinsic zincophilic properties. Subsequently, a charge transfer occurs between the carboxyl groups and Zn at the interface, causing the even local electric field, thereby attracting Zn^2+^ to migrate rapidly toward the interface and inducing their uniform deposition. In this process, the energy barrier for Zn^2+^ desolvation is lowered, and the strong adsorption of SUSA effectively excludes SO_4_^2−^ and H_2_O molecules from the interface, thus significantly suppressing deleterious side reactions.

The exceptional adsorption performance also profoundly influences inner kinetics behavior. The Zn^2+^ transference number test was operated to determine the effect of Zn^2+^ on the overall transference. The introduction of the SUSA additive results in an increase of this parameter from 0.26 to 0.41, lowering the concentration polarization in the electrolyte (Fig. 5h). Concurrently, the apparent activation energy calculated based on the Arrhenius formula decreases from 21.36 kJ mol^−1^ in the baseline electrolyte to 9.57 kJ mol^−1^ in the optimized electrolyte, reflecting a reduced macroscopic barrier to Zn^2+^ deposition (Figs. 5i and S30) [44, 45]. This might be attributed to the inherent zincophilic and water-repellent properties of SUSA, which contribute to the kinetic process of Zn^2+^ at the interface after reshaping the IHP structure. Also, it enables a rapid and stable shift from 2 to 3D Zn^2+^ diffusion in the ZSO/SUSA-0.02 electrolyte, thereby substantially improving Zn^2+^ deposition uniformity (Fig. 5j). Based on the cyclic voltammogram (CV) curves of Zn||Cu batteries, the addition of SUSA reduces the nucleation overpotential by 23 mV (Fig. 5k). The markedly lower nucleation overpotential observed in the ZSO/SUSA-0.02 electrolyte relative to ZSO under different current densities indicates a lowered energy barrier for nucleation (Figs. 5l and S31). This demonstrates strong adaptability under high-current–density conditions and favors the generation of a denser deposition morphology.

The response current is a key metric for assessing the reversibility of Zn deposition/dissolution reactions, kinetic rates, and interfacial stability within batteries. As shown in Fig. 5m, the enhanced response current is highly consistent with the reduced voltage hysteresis observed in the cycling of the ZSO/SUSA-based Zn||Zn symmetric cells. The above results confirm that in addition to the bolstered adsorption of SUSA achieving the improved uniformity of the electric field, it also regulates the ion concentration field at the interface. This synergistic enhancement of the interfacial field chemistry promotes the fast, stable, and dense deposition behavior of Zn^2+^ with a smaller nucleation overpotential, a lower desolvation energy barrier, and a more uniform Zn^2+^ flux distribution.

The interfacial kinetics throughout the electrochemical process were explored according to the distribution of relaxation times (DRT) obtained through in situ electrochemical impedance spectroscopy (EIS). The deposition in ZSO electrolyte manifests as pronounced, irregular peaks, suggesting frustrating kinetic contradiction. Conversely, the relatively uniform peak profile in ZSO/SUSA-0.02 electrolyte suggests low interfacial impedance and homogeneous deposition behavior (Fig. 5n, o) [46, 47]. As illustrated in Fig. 5p, the voltage spike in the ZSO electrolyte suggests that stripping occurs from the bulk anode rather than from the freshly deposited Zn. This process readily leads to dead Zn formation and lower anodic utilization efficiency [48]. By contrast, stable plating/stripping behavior is achieved with the ZSO/SUSA-0.02 electrolyte, once again confirming the overall kinetic-promoting effect of interfacial field chemistry regulation.

**Fig. 6:**
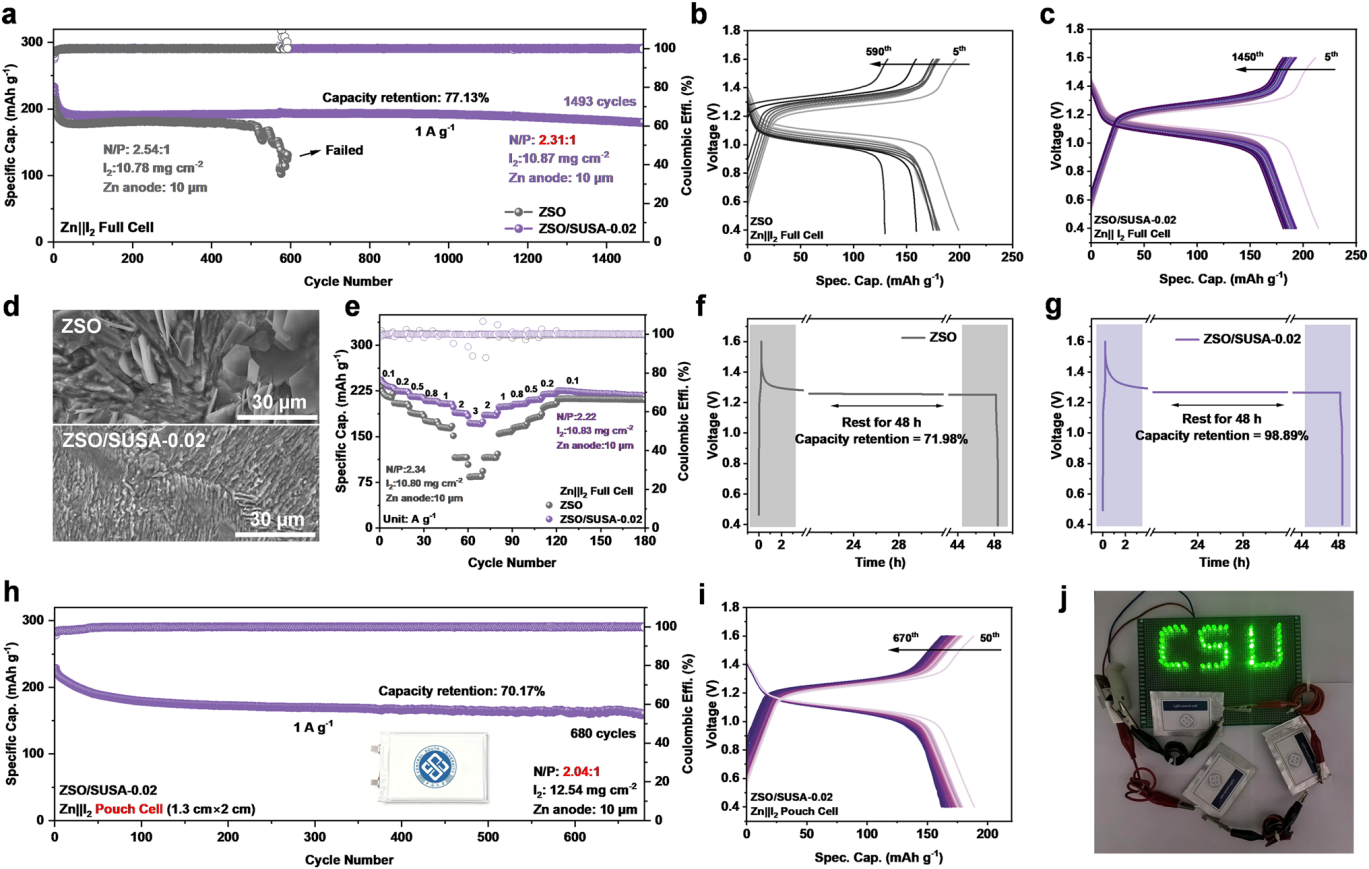
Exploration of the practical application of SUSA and electrochemical performance of Zn||I_2_ full cells. **a** Coin full cell cycling performance with similar N/P ratios in varying electrolytes at 1 A g^−1^ and corresponding voltage–capacity curves in **b** ZSO and **c** ZSO/SUSA-0.02 electrolytes. **d** Post-200-cycle Zn anode of Zn||I_2_ full cells within ZSO and ZSO/SUSA-0.02 electrolytes at 1 A g^−1^. **e** Rate performance of full cells with similar N/P ratios in different electrolytes. Self-discharge tests of full cells within **f** ZSO and **g** ZSO/SUSA-0.02 electrolytes. **h** The Zn||I_2_ pouch-cell (I_2_:1.3 cm × 2 cm) cycling performance in optimized electrolyte and **i** corresponding voltage-capacity curves at 1 A g^−1^. **j** Exhibition of LED lights powered by ZSO/SUSA-0.02-based pouch Zn||I_2_ cells

To evaluate the viability of SUSA in real use, full cells incorporating low N/P ratio with I_2_ cathodes were fabricated. As shown in Fig. 6a, the Zn (10 μm)||I_2_ coin full cell using ZSO/SUSA-0.02 sustains beyond 1493 cycles at 1 A g^−1^ with I_2_ at 10.87 mg cm^−2^, maintaining capacity retention of 77.13% with 179.86 mAh g^−1^ capacity, which markedly outpaces the cell matching I_2_ with similar masses within ZSO electrolyte. Of note is that during operation, the charge–discharge curves of ZSO-based full cell exhibit considerable dispersion, whereas those in ZSO/SUSA-0.02 electrolyte show excellent consistency (Fig. 6b, c), indicating the positive role of SUSA in stabilizing the capacity. Also, another full cell with the addition of ZSO/SUSA-0.02 electrolyte stably cycles over 1240 cycles under 0.5 A g^−1^, attaining a lower N/P ratio (1.99) and 80.10% capacity retention (Fig. S32). Under conditions mirroring the N/P ratio from the coin full cell in Fig. 6a, the post-200-cycle Zn anode in the ZSO electrolyte revealed arbitrary dendrite growth on its surface, yet the ZSO/SUSA-0.02-based electrode preserved a tightly packed deposition (Fig. 6d). Further, the rate capability was examined to verify the exceptional cycling stability and electrochemical reversibility. Notably, the severe capacity degradation observed in the ZSO electrolyte at elevated current densities underscores the inherent limitations posed by insufficient kinetics. In contrast, the cell incorporating the SUSA modification exhibits excellent capacity recovery even after high-current cycling at 3 A g^−1^ (Fig. 6e).

And resting tests were carried out to scrutinize self-discharge features. After a 48-h rest, the cell in ZSO/SUSA-0.02 retained an amazing capacity retention of 98.89%, compared to a mere 71.98% for the ZSO electrolyte, implying strong suitability of ZSO/SUSA-0.02 with the I_2_ cathode (Fig. 6f, g). Further, we measured the iodine content in electrolytes after different numbers of cycles at 1 A g^−1^. As shown in Fig. S33, after 50 cycles, a high concentration of iodine reaching 2244.75 mg L^−1^ in the ZSO electrolyte was detected by inductively coupled plasma mass spectrometer (ICP-MS) analysis. In contrast, the ZSO/SUSA-0.02 electrolyte remained clear and transparent after the same number of cycles, with lower iodine concentration (1197.26 mg L^−1^). Moreover, the dissolved iodine content increased by no more than 15% between the 50th and 100th cycles, suggesting that the iodine content reaches an equilibrium in the later stage, and the system enters a cycling steady state.

Consequently, a pouch-type full cell (I_2_: 1.3 cm × 2 cm) with limited Zn anode (10 μm) paired with high-mass-loading I_2_ (12.54 mg cm^−2^) was constructed to assess practicality validation. Despite an exceptionally limited N/P ratio of 2.04, the anticipated device retains 70.17% of its capacity (equivalent to 160.35 mAh g^−1^) after 680 cycles at 1 A g^−1^ (Fig. 6h). The highly convergent voltage-specific capacity profiles further confirm its stability and constant power output (Fig. 6i). Accordingly, three aforementioned pouch-type cells with series-connected status successfully energized the Central South University logo consisting of green LEDs (Fig. 6j). The successful application of full cells confirms the commercial potential of this strategy, providing a distinctive and efficient paradigm for interfacial field modulation toward high-power AZMBs.

## Conclusions

Overall, we present an interfacial field chemistry strategy via ultra-trace (0.36%) SUSA additive to engineer the IHP of Zn anodes, leveraging comprehensive characterization to elucidate the structure-performance relationship between additive adsorption and the significant enhancement of deep fast-charging conditions. Through the bolstered adsorption, SUSA molecules reshape the molecular/ion distribution field in the IHP, excluding H_2_O and SO_4_^2−^ as well as assisting in the homogenization of Zn^2+^ flux, thus mitigating parasitic reactions on the Zn surface. Concurrently, their charge-transfer interaction with the anode homogenizes the interfacial electric field, which facilitates rapid Zn^2+^ kinetics in the electrolyte. This strong synergistic adsorption reduces the Zn^2+^ desolvation energy barrier and nucleation overpotential, which collectively guide accelerated and even Zn deposition. In addition, the SUSA molecules in the EDL layer can also reconstruct the HB-network structure in the bulk electrolyte, significantly extending the lifespan of the battery system in a high-temperature environment of 60 °C (over 300 h). The system also has a wide service temperature range adaptability from − 20 to 60 °C. This multi-path effect of bolstered interfacial field chemistry endows the Zn anode with the ability to attain maximized utilization under deep-discharge and fast-charging conditions. Hence, the modified Zn||Cu asymmetric cells cycled at 2 mA cm^−2^ and 1 mAh cm^−2^ achieve an exceptional average CE of 99.48% (over 1600 cycles). The modified Zn||Zn symmetric batteries demonstrate remarkable stability under challenging conditions such as large current density (over 1600 h at 5 mA cm^−2^, 2 mAh cm^−2^, and over 675 h at 10 mA cm^−2^, 10 mAh cm^−2^) and high depth of discharge (56.9%). In practical terms, the modified Zn (10 µm) ||I_2_ full cell sustains over 1490 cycles with 77.13% capacity retention at 1 A g^−1^, achieving a harsh N/P ratio of 2.31. Furthermore, the Zn (10 µm) ||I_2_ (12.54 mg cm^−2^) pouch full cell exhibits stable operation for over 680 cycles with 70.17% capacity retention and an ultralow 2.04 N/P ratio. And such series-connected pouch-based cells demonstrated excellent capability by energizing the green LEDs. This work highlights the key role of interfacial field regulation in enabling ultrastable deep fast-charging Zn anodes and offers a universally instructive paradigm for rational interface design in other advanced energy storage technologies pursuing high-rate and high-utilization performance.

## Supplementary Information

Below is the link to the electronic supplementary material.

Supplementary file1 (DOCX 5177 KB)
