# Supplementary material for: Bolstered Interfacial Field Chemistry for Deep Fast-Charging Aqueous Zinc Metal Batteries
Source: Nanomicro Lett. 2026 Jul 29;19:8. doi: 10.1007/s40820-026-02314-5 (PMC13421562; doi:10.1007/s40820-026-02314-5)
Supplement: Supplementary file 1 — Supplementary file1 (DOCX 5177 KB) [file 40820_2026_2314_MOESM1_ESM.docx]

Supporting Information For

**Bolstered Interfacial Field Chemistry for Deep Fast-Charging Aqueous Zinc Metal Batteries**

Minxi Sun^1, #^, Yining Chen^1, #^, Congge Lu^1^, Jingkang Ma^1^, Qiuyuan Feng^2^, Shaoxing Li^1^, Tao Zhang^1^, Shuang Zhou ^1, *^ and Anqiang Pan ^1, 2, *^

^1^School of Materials Science and Engineering, Key Laboratory of Electronic Packaging and Advanced Functional Materials of Hunan Province, Central South University, Changsha 410083, P. R. China

^2^Xinjiang Key Laboratory of Advanced Metallic Materials Design and Application, Xinjiang Engineering Research Center of Environmental and Functional Materials, School of Materials Science and Engineering, Xinjiang University, Urumqi 830046, P. R. China

# Minxi Sun and Yining Chen contributed equally to this work.

* Corresponding authors. E-mail: zhoushuang2017@csu.edu.cn (Shuang Zhou); pananqiang@csu.edu.cn (Anqiang Pan)

**S1 Characterization Section**

***S1.1 Materials characterization***

The solutions prepared in this work were characterized by Fourier-transform infrared spectrometer (FTIR, Thermo Fisher Scientific Nicolet iN10), Raman microscope (Horiba LabRAM HR Evolution), and liquid-state nuclear magnetic resonance (NMR, BRUKER AVANCE 400). X-ray photoelectron spectroscopy (XPS) was performed on a Thermo Scientific K-Alpha spectrometer equipped with a monochromatic Al Kα X-ray source. The composition and structure of zinc anodes were examined by X-ray diffraction. Data were acquired on a Rigaku Mini Flex 600 diffractometer equipped with a Cu-Kα1 radiation source (λ = 1.5406 Å), scanning from 5° to 80° (2*θ*) at a rate of 10° per minute. The morphology of the Zn electrode was examined by field-emission scanning electron microscopy (SEM, Quanta FEG250). Its three-dimensional surface topography was analyzed using scanning laser confocal microscopy (SLCM, KEYENCE VK-X150). And the zinc plating process was monitored *in situ* using an optical microscope (Yuescope, YM530-YR). Electrode samples were obtained by dismantling Zn||Zn, Zn||I_2_ or Zn||Cu cells following a specified number of cycles under designated current densities. Once rinsed with DI water to eliminate remaining electrolyte and subsequently dried, the samples were moved to the apparatus for examining the Zn anode's or Cu foil’s morphology.

***S1.2 Electrochemical performance characterization***

Battery evaluations were performed via a NEWARE system (CT-4008-5 V-10/50 mA) employing CR2032-style coin batteries. Testing units were assembled under normal air conditions, each containing approximately 80 µL electrolyte. All separators, cutting to a diameter of 19 mm, has a 300 μm gauge and originates from Whatman™ Cytiva. The coulombic efficiency (CE) of zinc plating/stripping during cycling was determined using a Zn||Cu half-cell configuration, wherein a 15 mm diameter copper foil served as the cathode and a 12 mm diameter zinc foil (100 μm) functioned as the anode. Test batteries employed for depth-of discharge (DOD) were assembled using zinc foils (10 or 30 μm thick) punched into 15 mm diameter wafers. And for all symmetric cells (excluding DOD tested cells), zinc foils (100 μm thick) were punched into 12 mm diameter wafers. All full cells employed limited Zn foils (10 μm) as anodes, paired with pre-fabricated I_2_ electrodes. Zn||I_2_ coin full cells were assembled using 10 mm diameter I_2_ electrodes as the cathodes (areal loading: 10.78 - 12.98 mg cm^-2^) and 15 mm diameter zinc foils as the anodes. In the Zn||I_2_ pouch cells, the I_2_ cathode, Zn anode, Ti current collector and separator had dimensions of 13 × 20 mm, 16 × 23 mm, 19 × 24 mm and 23 × 30 mm, with a cathode areal loading of approximately 12.54 mg cm^-2^. During pouch cell assembly, aluminum-plastic laminates were employed as the external packaging. Heat sealing was initiated on three sides, followed by interior evacuation and final-side sealing to promote intimate contact among the internal components. Subsequently, a multimeter was utilized to measure the open-circuit voltage and confirm the absence of short-circuiting. Cyclic voltammetry (CV, 1 mV s^-1^ scan rate), linear sweep voltammetry (LSV, 1 mV s^-1^ scan rate), chronoamperometry (CA, at -200 mV overpotential), and response current (-0.35 V - 0.35 V, 1 mV s^-1^ of scan rate) measurements were conducted using a CHI660E electrochemical workstation. A three-electrode setup (Zn foil WE, Pt plate CE, Hg/Hg_2_Cl_2_ RE) was employed for tafel curve assessments on a CHI660E workstation. Differential Scanning Calorimetry (DSC) was conducted by Mettler DSC3 using liquid nitrogen for cooling. Zeta potential data were acquired using a Malvern Zetasizer Nano ZS90 instrument. Suspension samples were created throughn ultrasonication of electrodeposited Zn layers (1 mA cm^-2^, 5 mAh cm^-2^) on Zn foils in electrolytes, resulting in even distributions. Zn||Zn symmetric cells underwent in-situ electrochemical impedance spectroscopy (EIS) testing under galvanostatic cycling (1 mA cm^-2^), with data captured every 200 s. Inductively Coupled Plasma Mass Spectrometry (ICP-MS) was conducted by Agilent7700.

***S1.3 Theoretical computation section***

***S1.3.1 Molecular electrostatic potential and adsorption energy***

Based on the density functional theory (DFT), a computational method of quantum mechanics, the electrostatic potential and binding energy were calculated using the Materials Studio 2023 software. Specifically, the generalized gradient approximation (GGA) functional and Perdew-Burke-Ernzerhof (PBE) basis set were selected for the calculation. All molecular structures were optimized at the GGA/PBE level, followed by the calculation of the molecular electrostatic potential.

***S1.3.2 Frontier orbitals, differential charges and adsorption energy***

First-principles calculations were conducted using density functional theory (DFT). The exchange-correlation interactions were modeled using the Perdew-Burke-Ernzerhof (PBE) generalized gradient approximation (GGA). A plane-wave basis set a 450 eV energy cutoff was utilized for valence electron wavefunction expansion, and Brillouin zone integration was performed using a Γ-centered 4 × 4 × 1 k-point grid. Structural optimizations were performed with convergence criteria of 10^−5^ eV for total energy and 0.01 eV Å^-1^ for atomic forces. To eliminate periodic interactions, a 20 Å vacuum layer was implemented along the out-of-plane (z-axis) direction and the (6×4) cells were constructed to model the Zn (101) surface. Structural visualizations were generated using the VESTA software package. The adsorption energy was determined according to the following procedure:

$\text{E}_{\text{ads}}=\text{E}_{\text{all}}-\text{E}_{\text{system}}-\text{E}_{\text{ions}}$ (S1)

E_all_, E_system_ and E_ions_ represent the system's total energy, the bare substrate energy, and the free ion (molecule) energy, respectively, for adsorption scenarios with/without Zn atoms.

***S1.4 Calculation formulas***

***S1.4.1 Calculation of additive***

Given that the sulfosuccinate additive is supplied as an aqueous solution, the mass calculation for the additive refers to the solute itself, not the total solution mass. Therefore, the calculation formula is as follows:

$\text{m}_{\text{SUSA}}\text{=}\frac{\text{V×c}\text{×}\text{M}_{\text{SUSA}}}{\text{wt}\text{ \% in }\text{H}_{\text{2}}\text{O}}$ (S2)

Here, V is the total volume of the electrolyte, c stands the molar concentration of the target additive, and M signifies its molar mass.

***S1.4.2 Calculation of EDLC***Following earlier studies, EDLC values were computed via the expression:

***$C_{EDL}=\frac{\text{i}}{\text{v}}$*** (S3)

In this, C_EDL_ stands for capacitance, and i signifies the current deviation at the scan voltage midpoint for each scan rate.

***S1.4.3 Calculation of capacity of the inner Helmholtz layer (C_H_)*** [S1]

2 M ZnSO_4_ was substituted with 1 M Na_2_SO_4_ to eliminate Faraday current influence. LSV test was executed to pinpoint the voltage span for EDL capacitance evaluation. Minimal current from -1.032 to -1.35 V signified an EDL zone absent of charge/discharge. Specifically, EDL features a diffusion layer (DL) and compact layer (CL), with the CL containing inner/outer Helmholtz layer (IHP/OHP). Typically, OHP capacitance is overlooked given its substantial dielectric value, enabling the double-layer capacitance representation as:

$\frac{1}{\text{C}_{\text{d}}}=\frac{1}{\text{C}_{\text{H}}}+\frac{1}{\text{C}_{\text{D}}}$ (S4)

In which C_d_, C_H_ and C_D_ indicate capacity of EDL, IHP and DL.

The C_d_ can be determined through electrochemical impedance spectroscopy, and subsequently converted in accordance as:

$\text{C}_{\text{d}}=-\frac{1}{2\text{πfZ}_{\text{im}}}$ (S5)

In which f (Hz) stands for the impedance test frequency (10 Hz). Z_im_ (Ω) indicates the imaginary impedance component. With DL capacitance (C_D_) treated as invariant, IHP changes can be derived as follows:

$\frac{1}{\text{C}_{\text{d}}^{\text{''}}}-\frac{1}{\text{C}_{\text{d}}^{\text{'}}}=\frac{1}{\text{C}_{\text{H}}^{\text{''}}}-\frac{1}{\text{C}_{\text{H}}^{\text{'}}}$ (S6)

Wherein, C’_H_ shows negligible variation with potential and can therefore be treated as a constant (X). Substituting this value into the equation gives:

$\frac{1}{\text{C}_{\text{H}}^{\text{''}}}-\text{X}=2\text{πf}(\text{Z}_{\text{im}}^{\text{'}}-\text{Z}_{\text{im}}^{\text{''}})$ (S7)

Z’_im_ and Z’’_im_ indicate the imaginary impedance portions in Na₂SO_4_ and Na₂SO₄/SUSA solutions, respectively. Thus, shifts in capacitance echo IHP modifications under potential influenced by SUSA. With H_2_O dipoles dominating the IHP in conventional ZnSO₄ electrolyte, C_d_ exhibits negligible fluctuation over the voltage span. On the contrary, the incorporation of SUSA effectively expels water molecules from the IHP layer, resulting in a pronounced fluctuation in the C_d_ value.

***S1.4.4 DOD calculation formula***

DOD, which denotes the proportion of actual zinc usage relative to the theoretical total provided zinc capacity and functions as the most dependable indicator for assessing anode efficiency, is computed via the following equation:

$\text{DOD}=\frac{\text{C}_{\text{pr}}}{\text{C}_{\text{th Zn}}\times\text{θ}}\times\text{100\%}$ (S8)

Where C_pr_ denotes the applied areal capacity (mAh cm^-2^), C_th Zn_ represents the theoretical capacity of Zn (5855 mAh cm^-3^), and *θ* indicates the thickness (cm) of zinc foil.

***S1.4.5 Calculation of Zn²⁺ transference number***

The Zn²⁺ transference number ($t_{{Zn}^{2+}}$) was assessed via EIS coupled with CA test under a 10 mV polarization voltage and 4000 s duration, and derived from the equation:

$\text{t}_{\text{Ζn}^{2+}}=\frac{\text{I}_{\text{s}}\text{(Δ}V-\text{I}_{0}\text{R}_{0}\text{)}}{\text{I}_{0}\text{(Δ}V-\text{I}_{\text{S}}\text{R}_{\text{S}}\text{)}}$ (S9)

In which I_0_ and I_s_ indicate starting and equilibrium currents, whereas R_0_ and R_s_ signify the resistances prior to and following the CA measurement. ΔV corresponds to the imposed polarization voltage (10 mV).

***S1.4.6 Calculations of the activation energy***

According to Arrhenius formula calculates:

$\frac{1}{R_{ct}}=Aexp(-\frac{E_{a}}{RT})$ (S10)

In which R_ct_ represents the charge-transfer resistanc, A represents a coefficient, E_a_ represents the activation energy, R represents the universal gas constant, and T represents the temperature in Kelvin.

***S1.4.7 Calculation of N/P ratio***

N/P ratio serves as a pivotal metric for evaluating the viability of AZMBs. A low N/P ratio is essential for achieving the high energy density battery system, as it maximizes zinc utilization, which is a key requirement for practical AZIBs with high cathode loading and lean electrolyte. The specific calculation formula for the N/P ratio is as follows:

$\text{N}/\text{P}=\frac{\text{C}_{\text{negative}}}{\text{C}_{\text{positive}}}=\frac{\text{θ}\times\text{C}_{\text{th Zn}}}{\text{m}\times\text{C}_{\text{I}_{2}}}$ (S11)

Where C_negative_ represents the areal capacity of the anode (mAh cm^-2^), calculated as the product of thickness of zinc anode (*θ*, cm) and theoretical capacity of Zn (C_th Zn_, 5855 mAh cm^-3^). And C_positive_ represents the areal capacity of the cathode (mAh cm^-2^), calculated as the product of active anode material loading of cathode (g cm^-2^) and maximum specific capacity of the cathode (mAh g^-1^).

**S2 Supporting Figures**


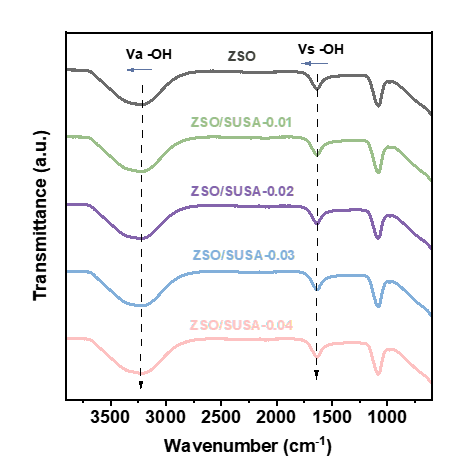


**Fig. S1** FTIR spectral analysis of ZSO, ZSO/SUSA-0.01, ZSO/SUSA-0.02, ZSO/SUSA-0.03 and ZSO/SUSA-0.04 electrolytes.


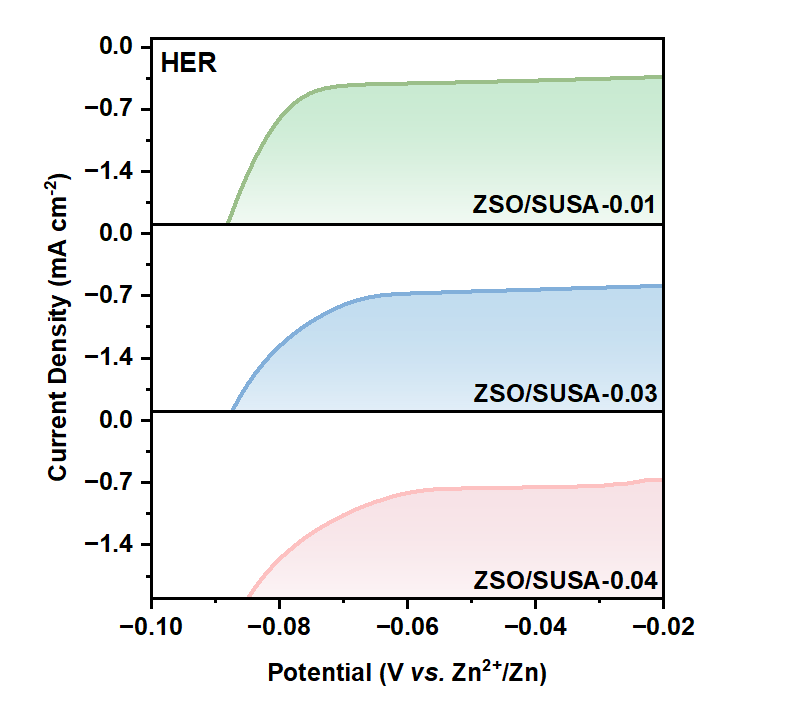


**Fig. S2** LSV profiles of ZSO/SUSA-0.01, ZSO/SUSA-0.03 and ZSO/SUSA-0.04 electrolytes.


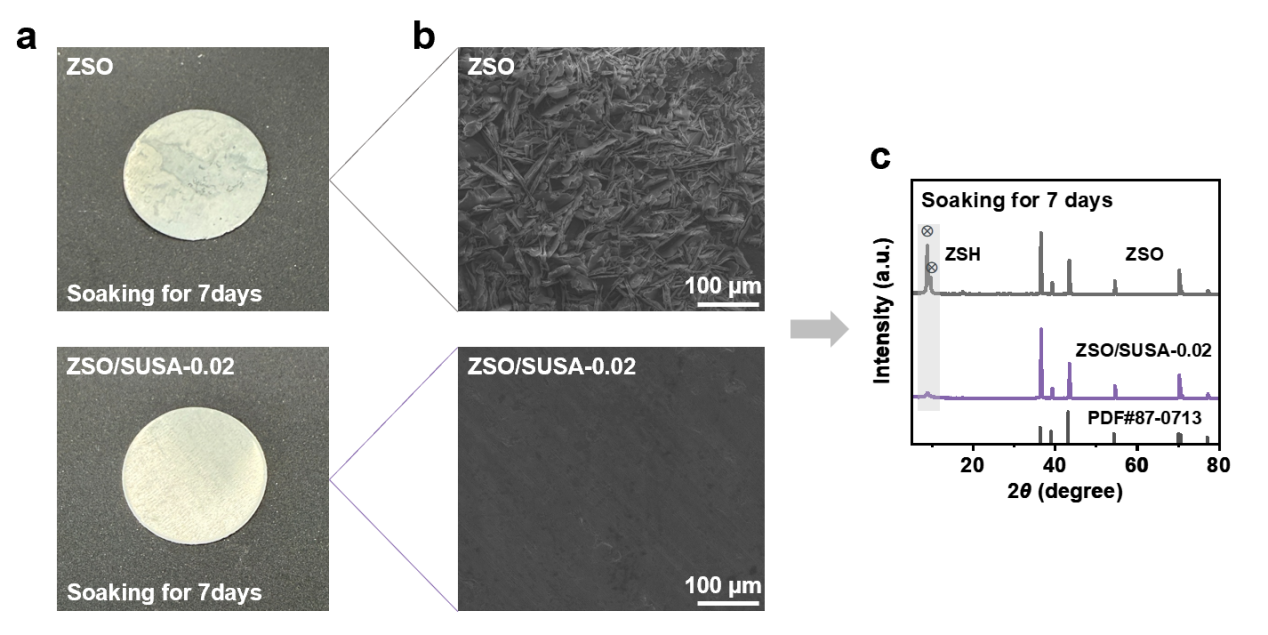


**Fig. S3** **a** Optical photographs, **b** SEM images and **c** XRD patterns of Zn foils after being soaked in ZSO and ZSO/SUSA-0.02 electrolytes for 7 days.

Analysis of the XRD patterns reveals the presence of two pronounced reflections, located at 8.82° and 9.66°. These are identified as the signature peaks of Zn_4_SO_4_(OH)_6_·4H_2_O (PDF#44-0673) and Zn_4_SO_4_(OH)_6_·3H_2_O (PDF#39-0689), respectively.


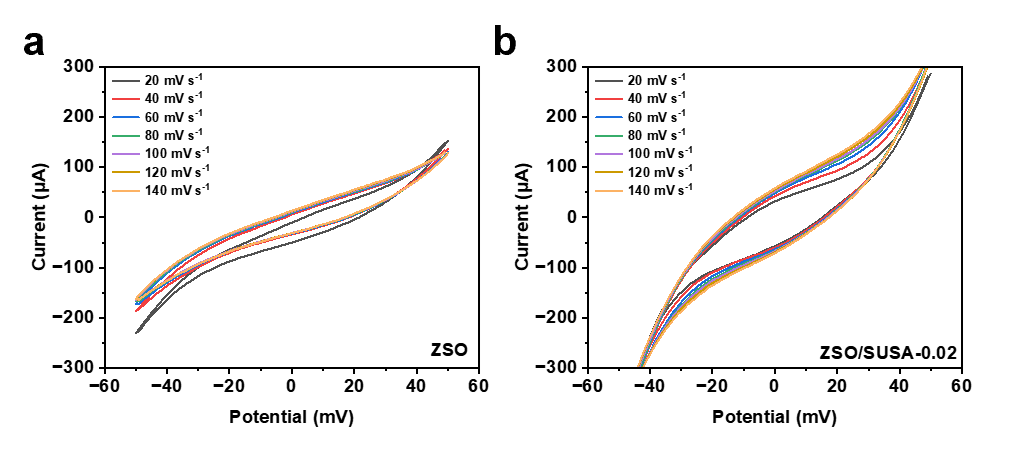


**Fig. S4** Cyclic voltammograms acquired at varying scanning rates for electrochemical double-layer capacitance assessment within **a** ZSO and **b** ZSO/SUSA-0.02 electrolytes.


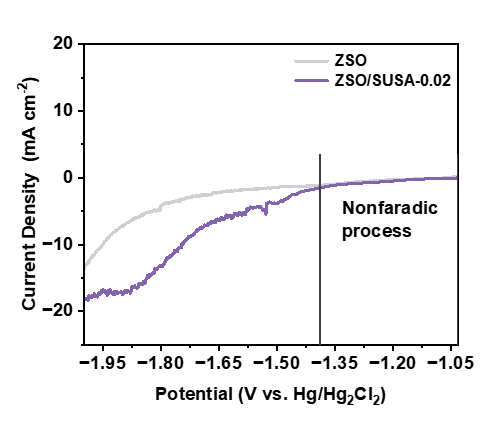


**Fig. S5** LSV with and without SUSA tested in a three-electrode system.


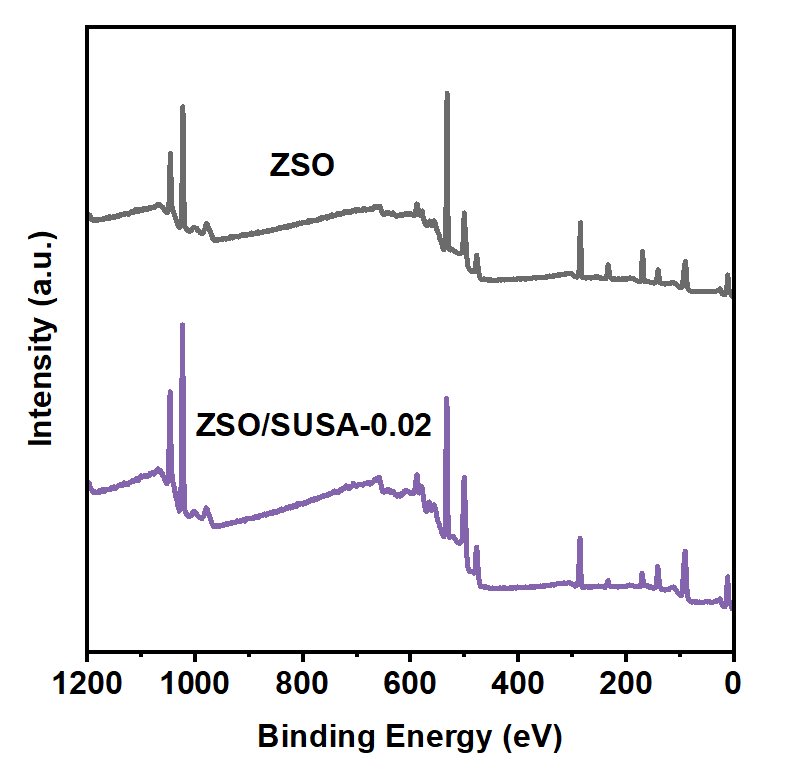


**Fig. S6** X-ray photoelectron spectroscopy (XPS) survey spectrum of of zinc anode electroplated for 5 min at 1 mA cm^-2^ within electrolytes with/without SUSA.


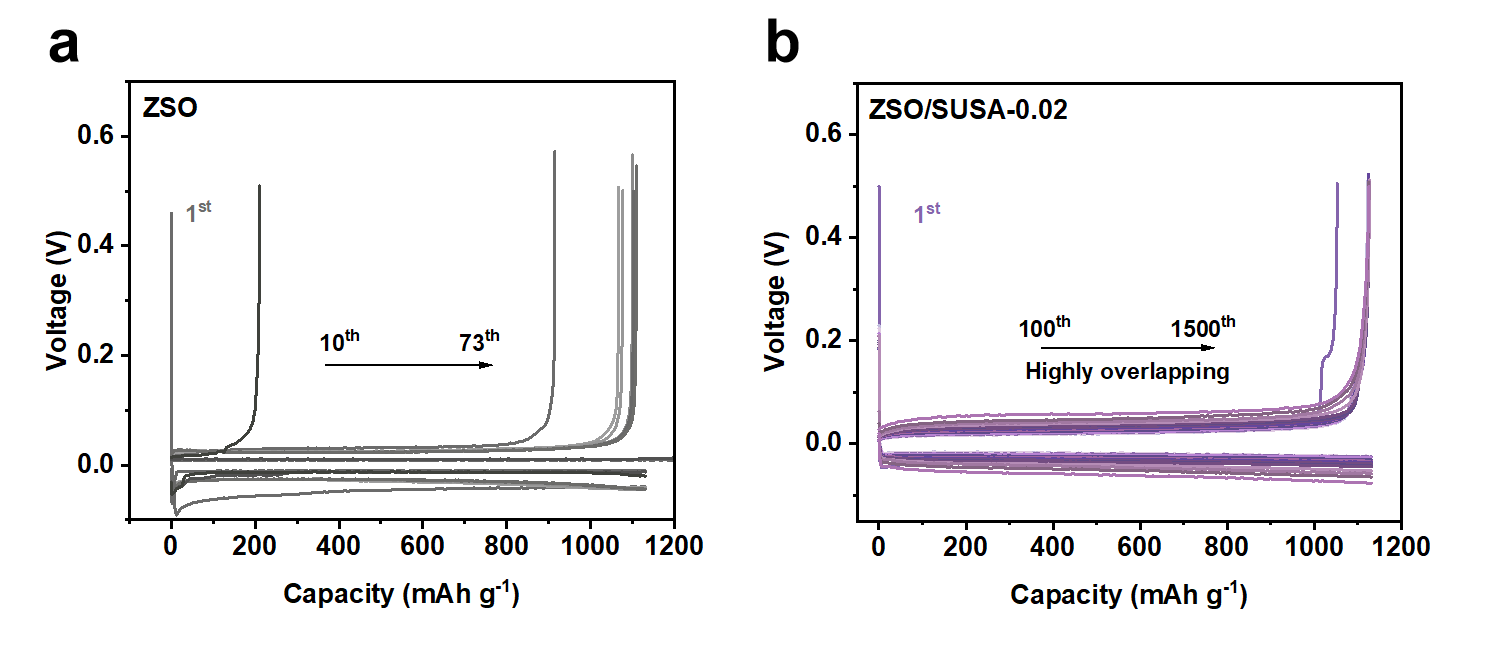


**Fig. S7** Voltage profiles of Zn||Cu half-cells cycled within **a** ZSO and **b** ZSO/SUSA-0.02 electrolytes.


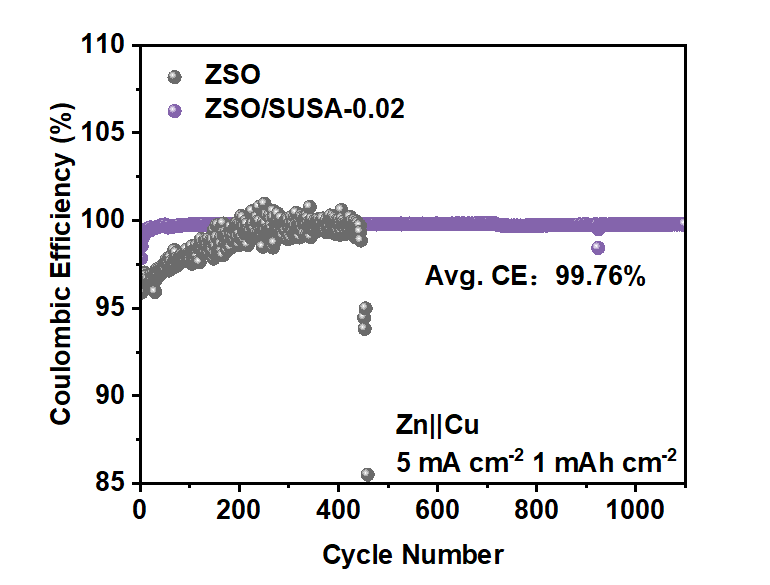


**Fig. S8** CE of Zn||Cu half cells in ZSO with/without SUSA electrolytes cycled under 5 mA cm^-2^ and 1 mAh cm^-2^.


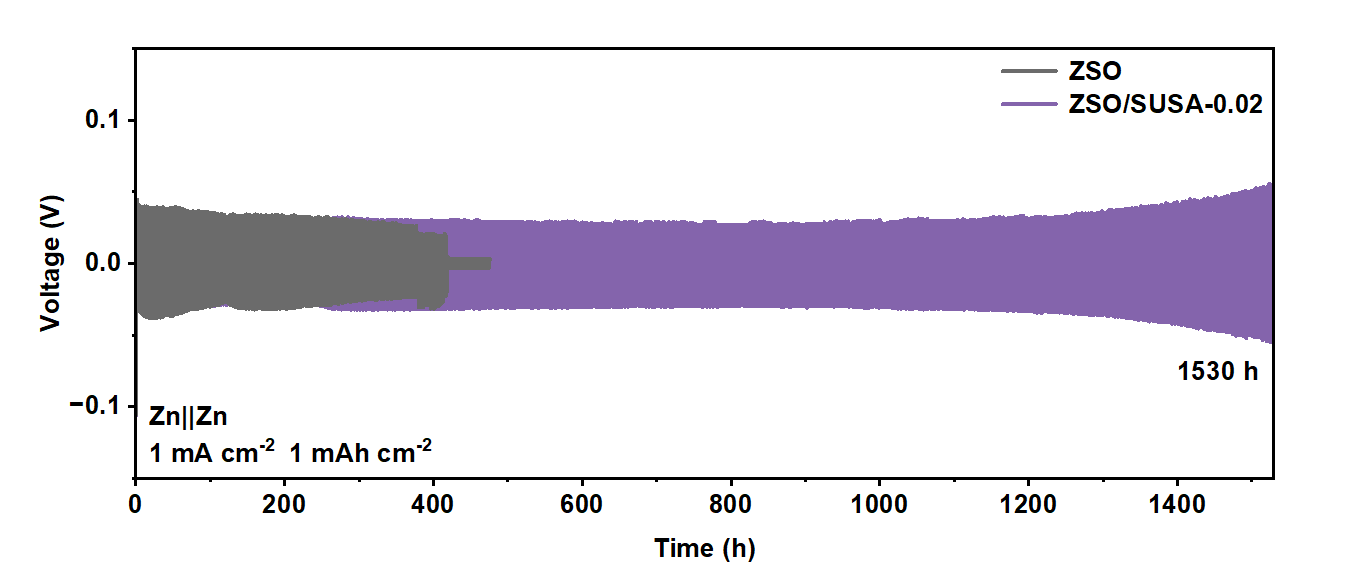


**Fig. S9** Cycling performance characterization of Zn||Zn symmetric cells in ZSO with/without SUSA electrolytes under 1 mA cm^-2^ and 1 mAh cm^-2^.


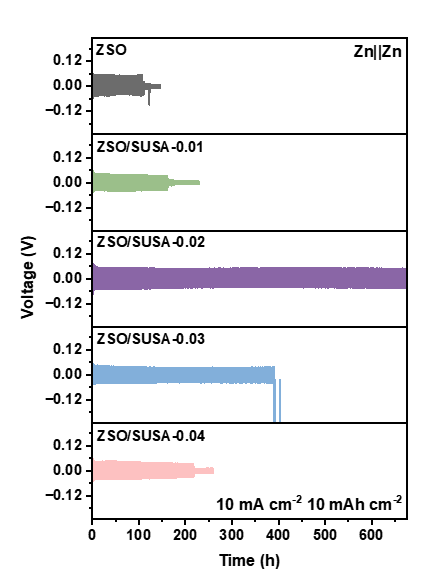


**Fig. S10** Cycling performance characterization of Zn||Zn symmetric cells in ZSO with/without SUSA electrolytes under 10 mA cm^-2^ and 10 mAh cm^-2^.


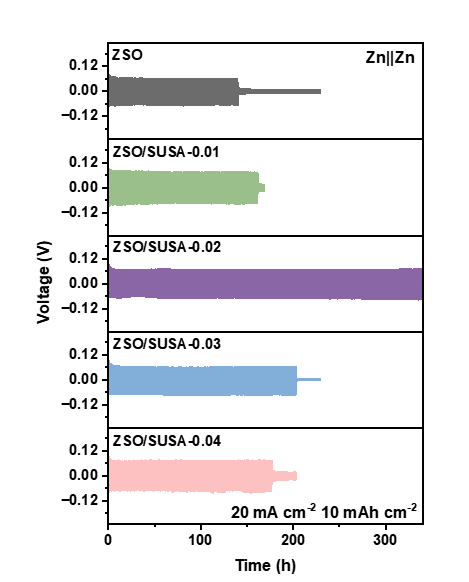


**Fig. S11** Cycling performance characterization of Zn||Zn symmetric cells in ZSO with/without SUSA electrolytes under 20 mA cm^-2^ and 10 mAh cm^-2^.


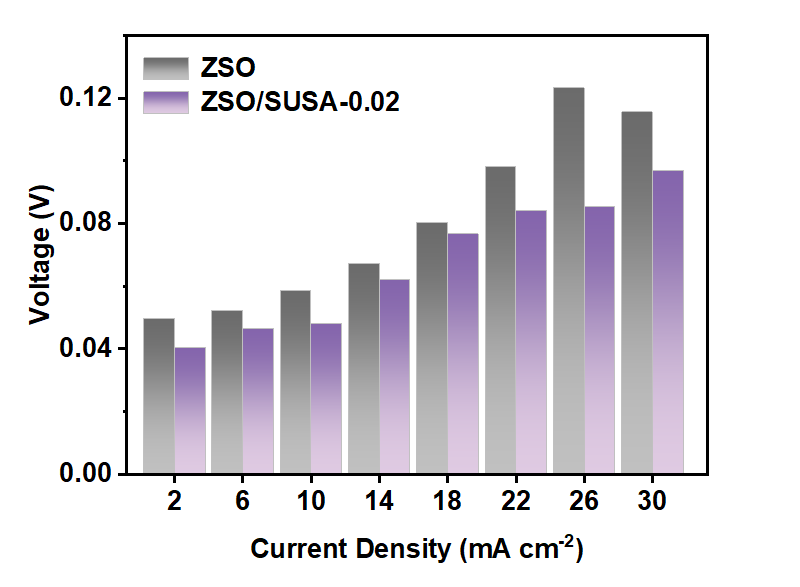


**Fig. S12** Comparison of polarization voltages of Zn||Zn symmetric cells within different electrolytes under rate performance tests.


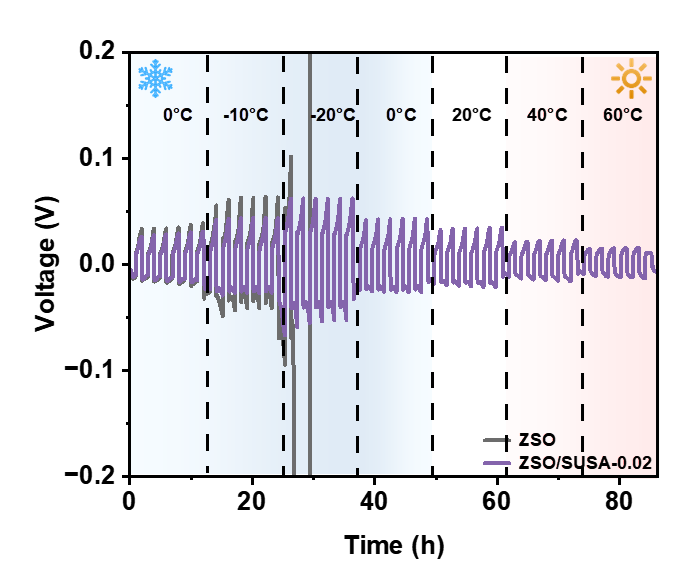


**Fig. S13** Rate performance tests under varying temperatures within ZSO electrolytes with/without SUSA adding.


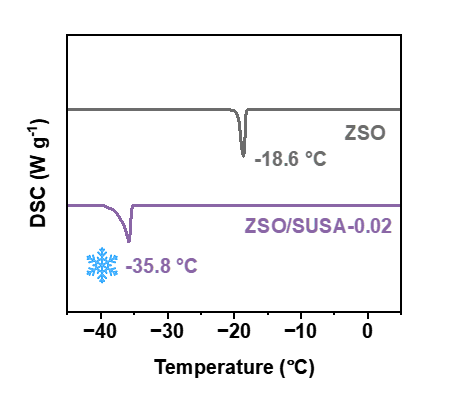


**Fig. S14** DSC test from 10 to -45 ℃ at a freezing rate of 5 ℃ min^-1^.


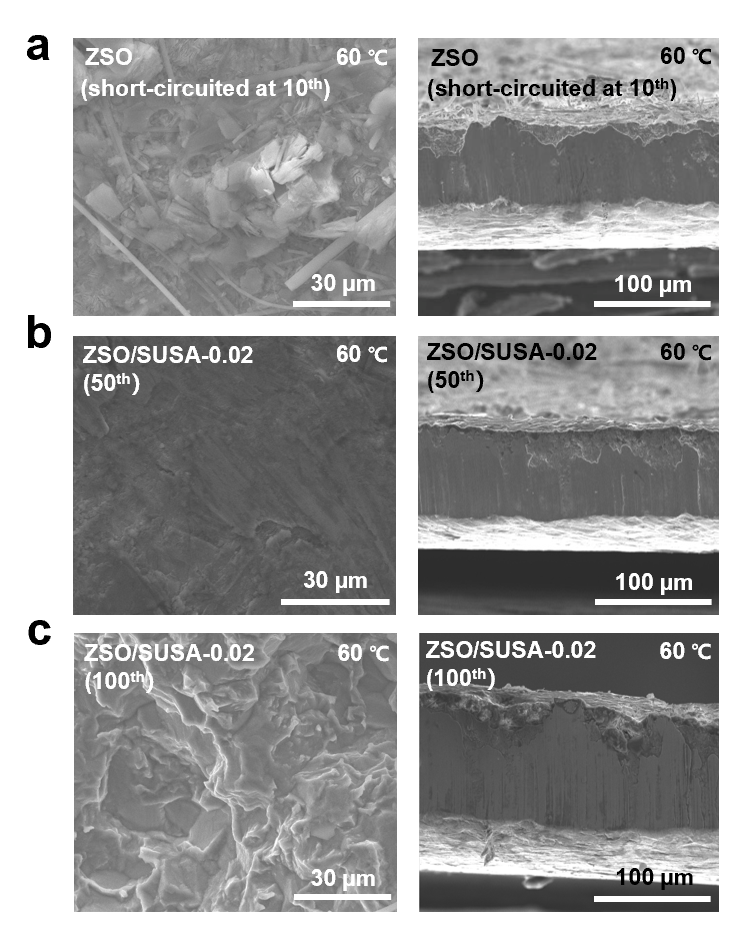


**Fig. S15** SEM images of the frontal and cross-sectional views of the Zn electrode at 0.25 mA cm^-2^ and 0.25 mAh cm^-2^ under 60 ℃ in **a** ZSO electrolyte (short-circuited at the 10^th^ cycle), **b** ZSO/SUSA-0.02 electrolyte after 50 cycles, **c** ZSO/SUSA-0.02 electrolyte after 100 cycles.


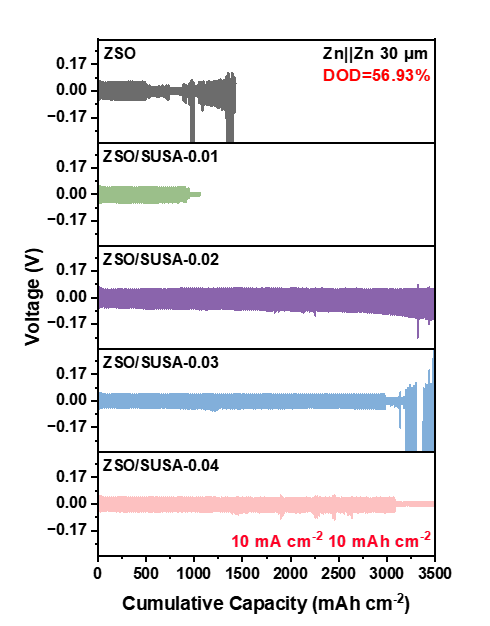


**Fig. S16** Electrochemical performance characterization of Zn||Zn symmetric cells in ZSO with/without SUSA electrolytes cycled under 10 mA cm^-2^ and 10 mAh cm^-2^ (DOD≈56.93%).


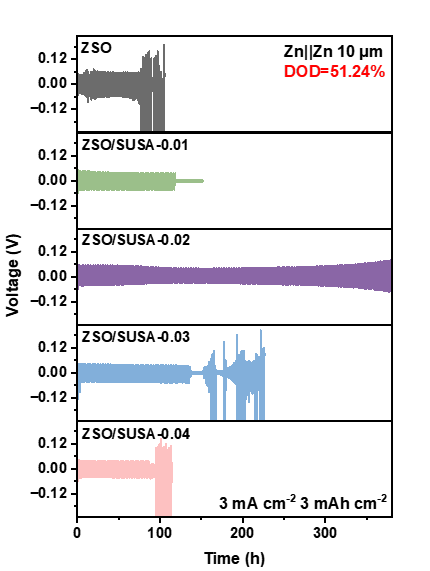


**Fig. S17** Cycling performance of Zn||Zn symmetric cells under DOD of 51.24%.


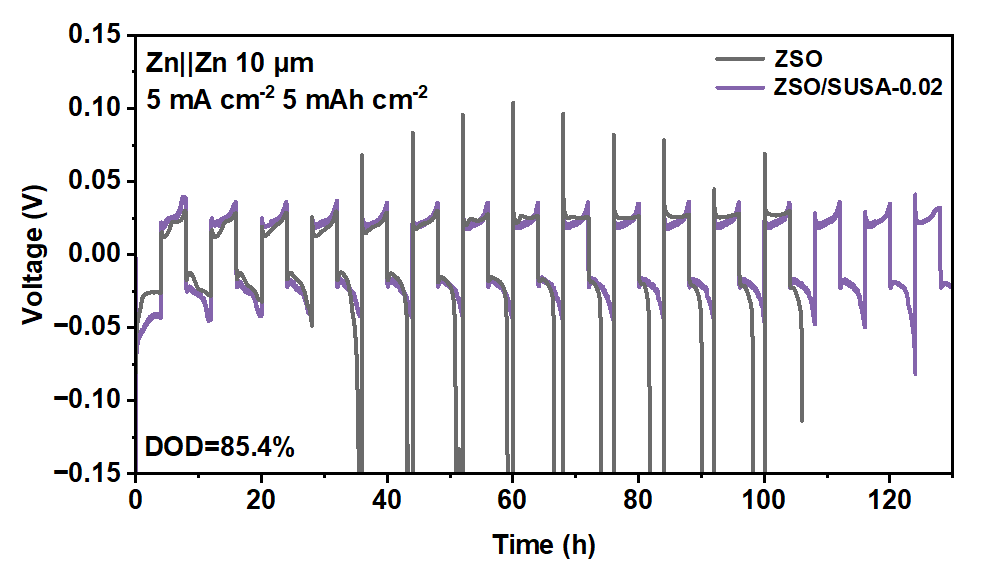


**Fig. S18** Cycling performance of Zn||Zn symmetric cells under DOD of 85.4%.


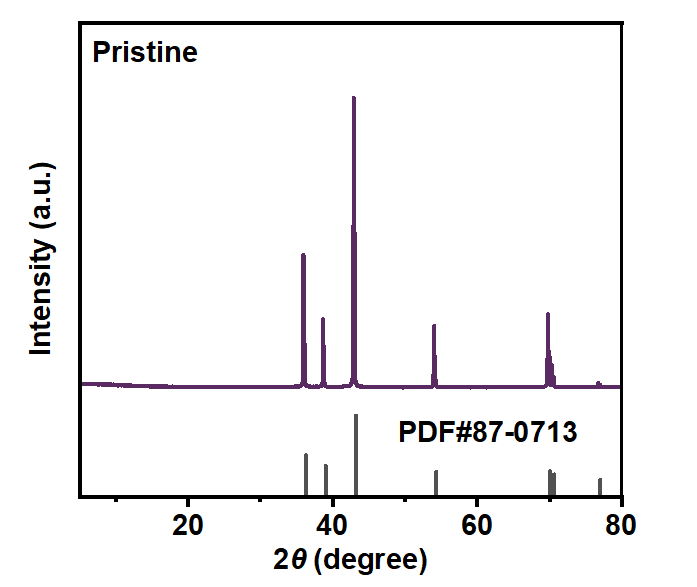


**Fig. S19** XRD pattern of pristine zinc.


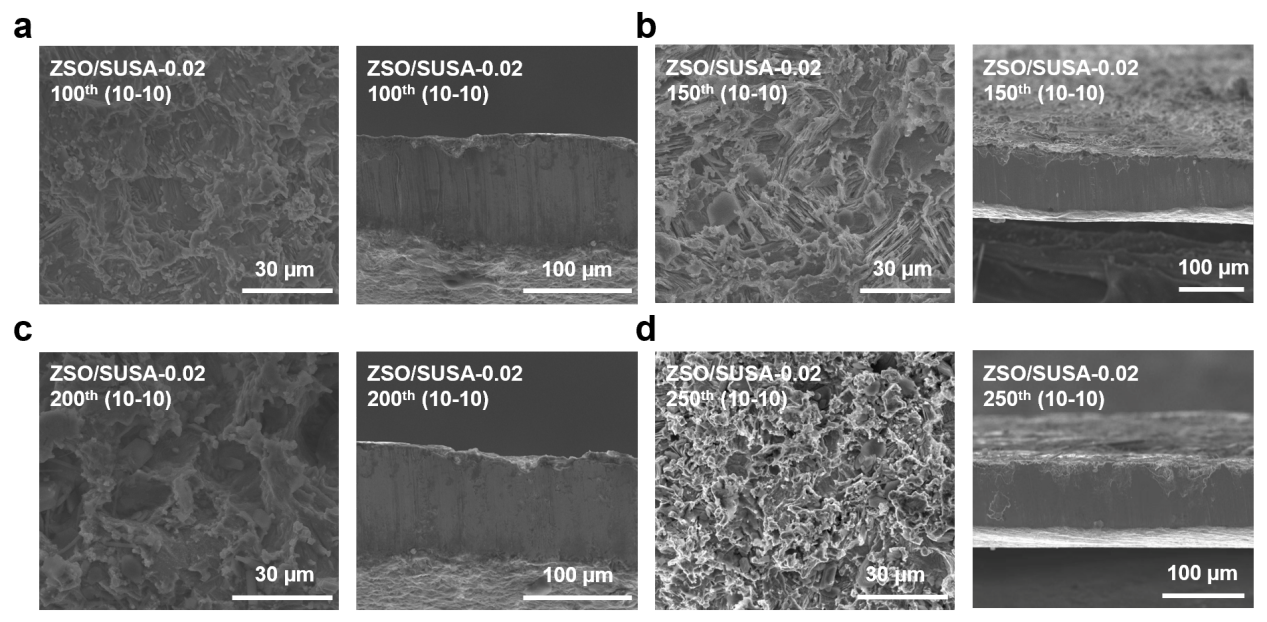


**Fig. S20** SEM images of Zn anodes after **a** 100, **b** 150, **c** 200, and **d** 250 cycles in ZSO/SUSA-0.02 electrolyte at 10 mA cm⁻² and 10 mAh cm⁻².


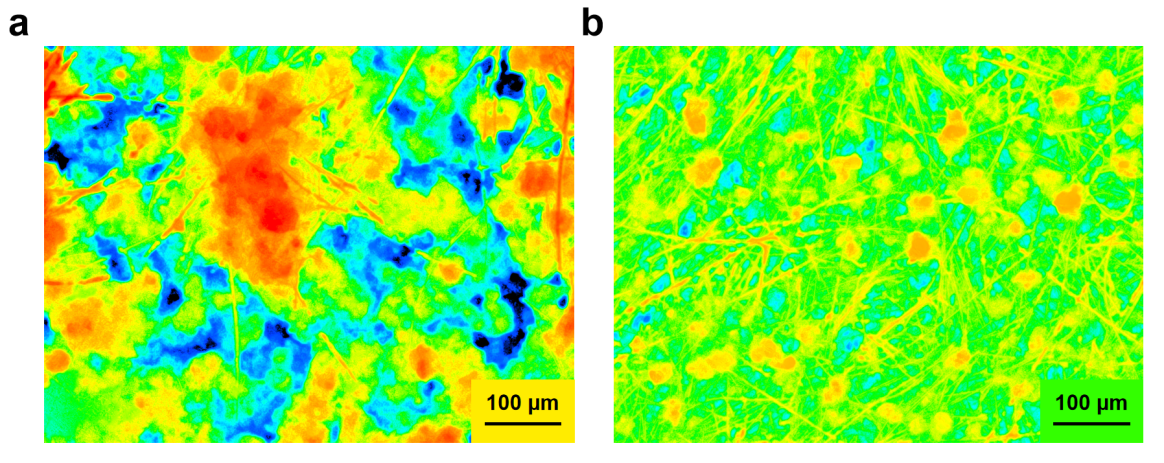


**Fig. S21** The LSCM images of Zn anodes cycled within **a** ZSO and **b** ZSO/SUSA-0.02 electrolytes at 10 mA cm^-2^, 10 mAh cm^-2^ after 50 cycles.


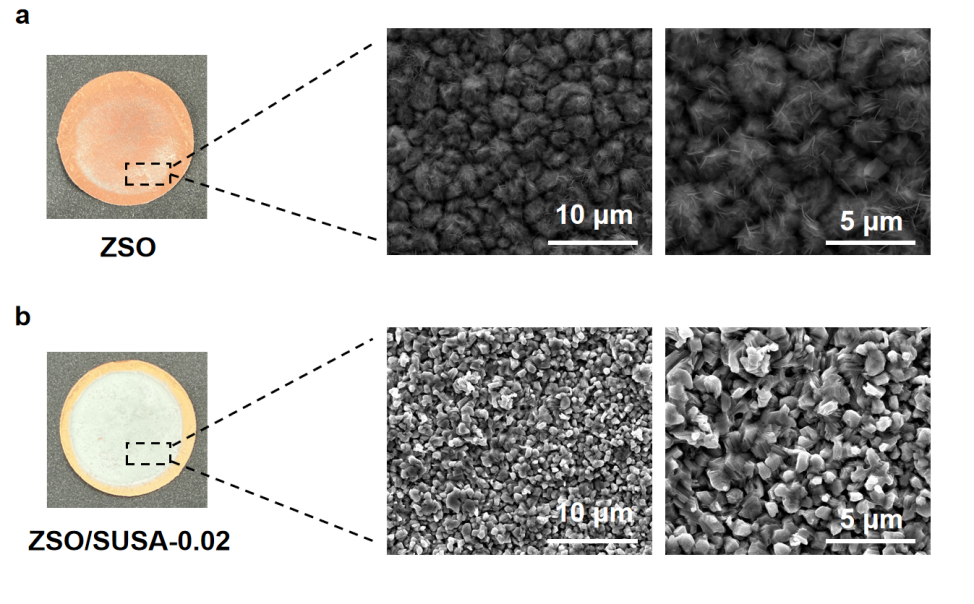


**Fig. S22** Optical photographs and SEM pictures of Cu foils when deposited under 2 mA cm^-2^, 1 mAh cm^-2^ within **a** ZSO and **b** ZSO/SUSA-0.02 electrolytes.


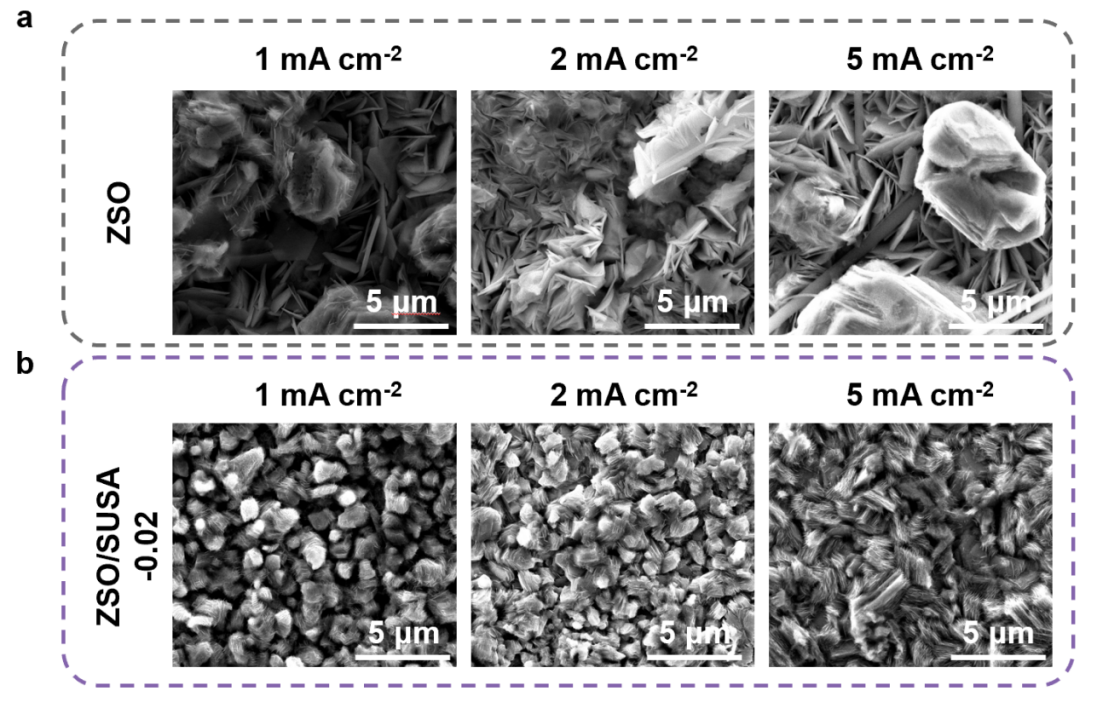


**Fig. S23** SEM pictures of Cu foils when deposited under 1 mAh cm^-2^ at various current densities of 1 mA cm^-2^, 2 mA cm^-2^ and 5 mA cm^-2^ respectively within **a** ZSO, **b** ZSO/SUSA-0.02.


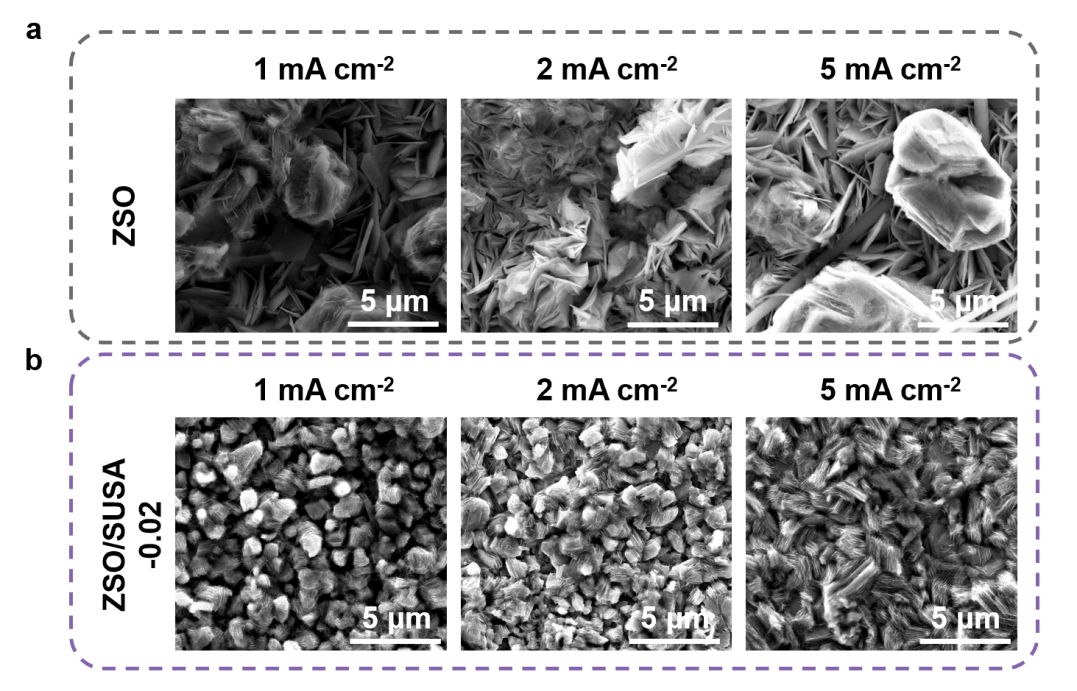


**Fig. S24** SEM pictures of Ti foils when deposited under 1 mAh cm^-2^ at various current densities of 1 mA cm^-2^, 2 mA cm^-2^ and 5 mA cm^-2^ respectively within **a** ZSO, **b** ZSO/SUSA-0.02.


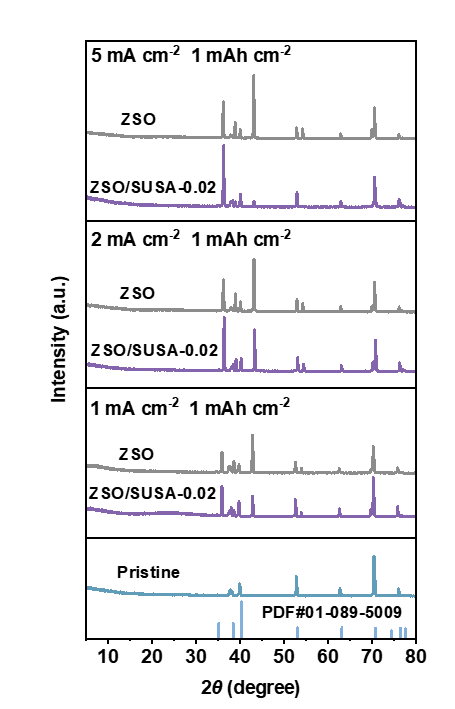


**Fig. S25** XRD patterns of Ti foils when deposited under 1 mAh cm^-2^ at various current densities of 1 mA cm^-2^, 2 mA cm^-2^ and 5 mA cm^-2^ respectively within ZSO electrolytes with/without SUSA adding.


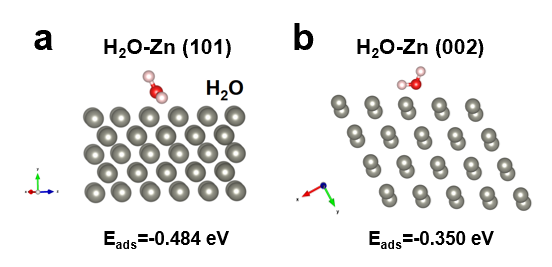


**Fig. S26** The adsorption energy of H_2_O on the **a** Zn (101) facet and **b** Zn (002) facet.


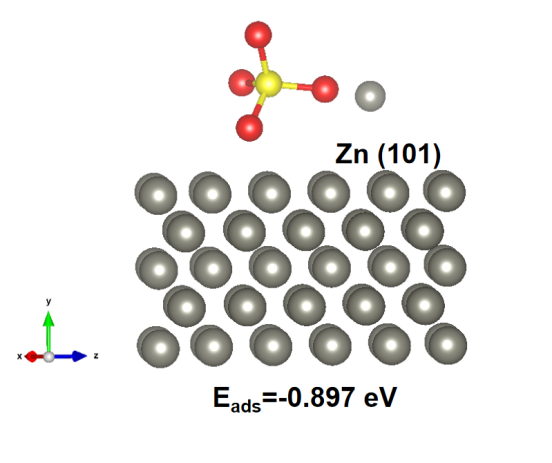


**Fig. S27** The adsorption energy of ZnSO_4_ on the Zn (101) facet.


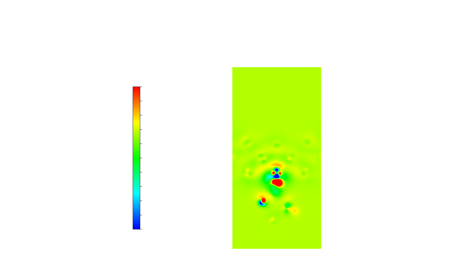


**Fig. S28** 2D of differential charge density plots of SUSA on Zn (002) facet.


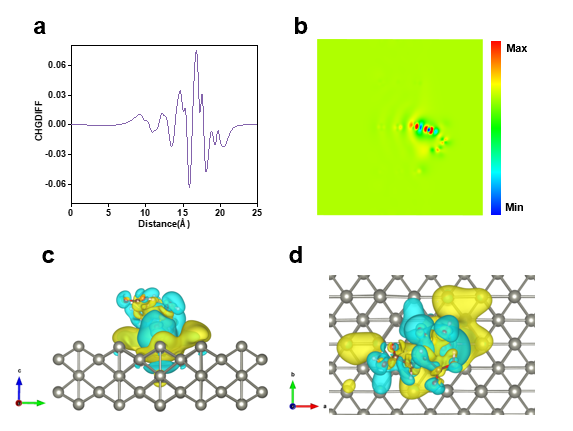


**Fig. S29** The **a** 1D and **b** 2D of differential charge density plots of SUSA on Zn (101) facet. The differential charge density plots of SUSA on Zn (101) facet from **c** front aspect and **d** top aspect.


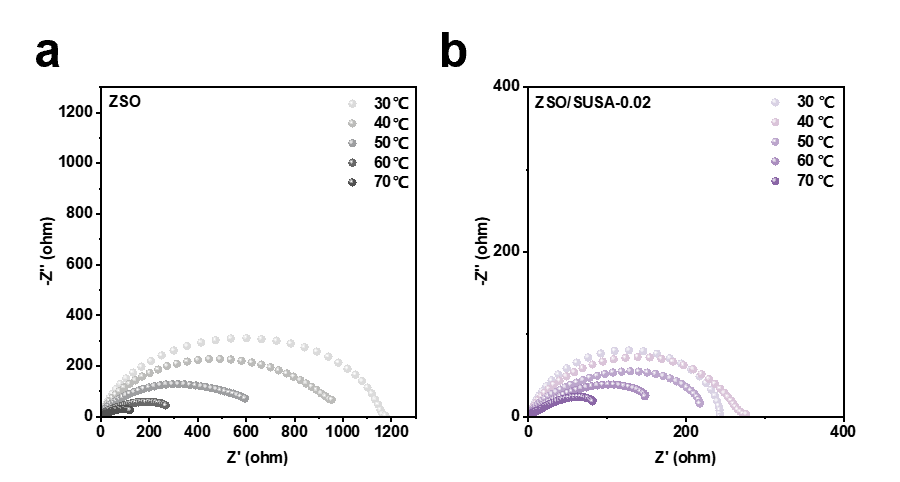


**Fig. S30** Nyquist plots recorded for Zn||Zn symmetric cells with **a** ZSO and **b** ZSO/SUSA-0.02 electrolytes at different temperatures.


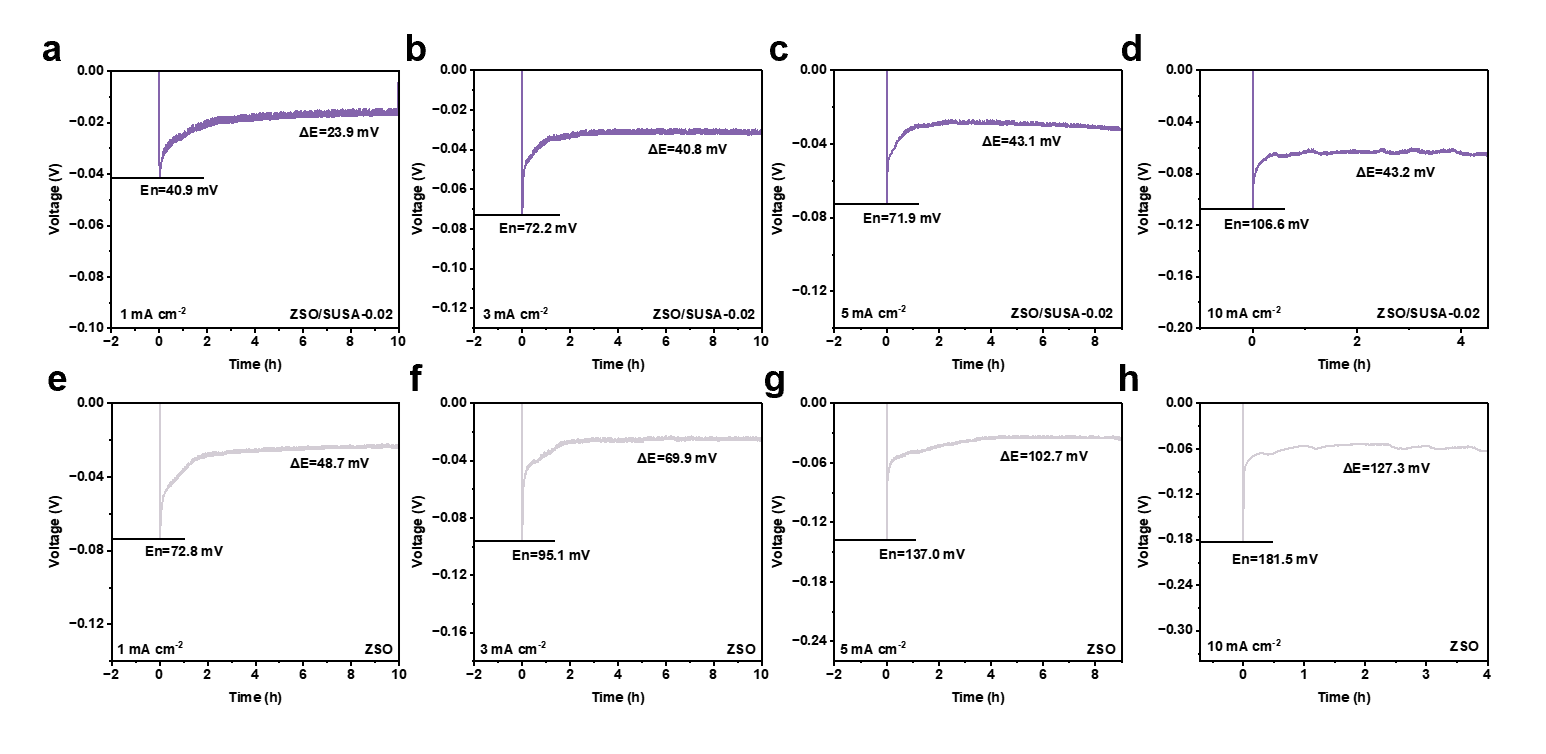


**Fig. S31** The galvanostatic nucleation overpotential (NOP) of Zn||Cu half-cells in different electrolytes under **a,e** 1 mA cm^-2^, **b,f** 3 mA cm^-2^, **c,g** 5 mA cm^-2^, **d,h** 10 mA cm^-2^.


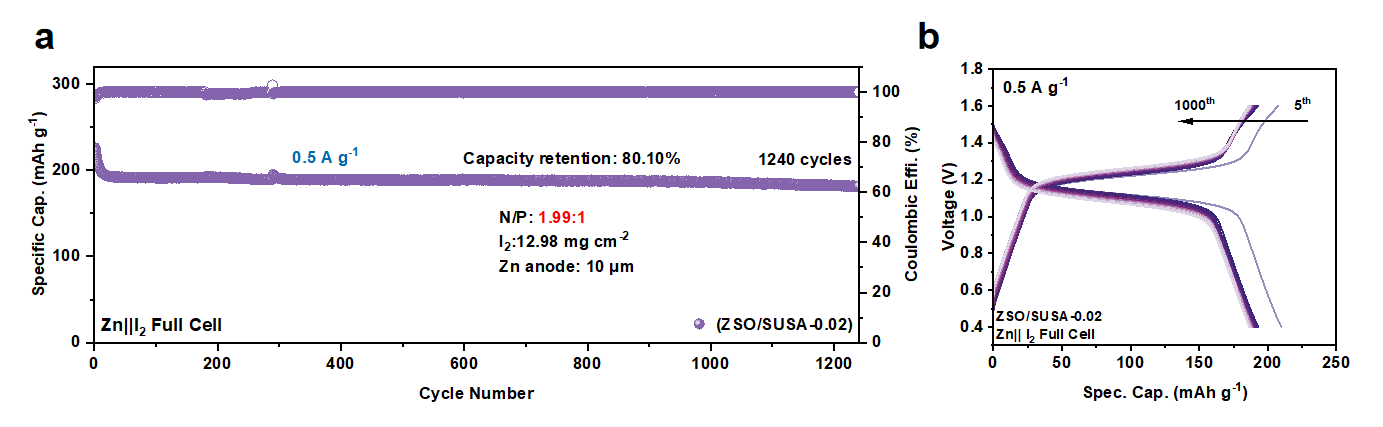


**Fig. S32 a** the coin full-cell cycling performance within ZSO/SUSA-0.02 electrolyte at 0.5 A g^-1^ with a low N/P ratio of 1.99 and **b** corresponding voltage-capacity curves.


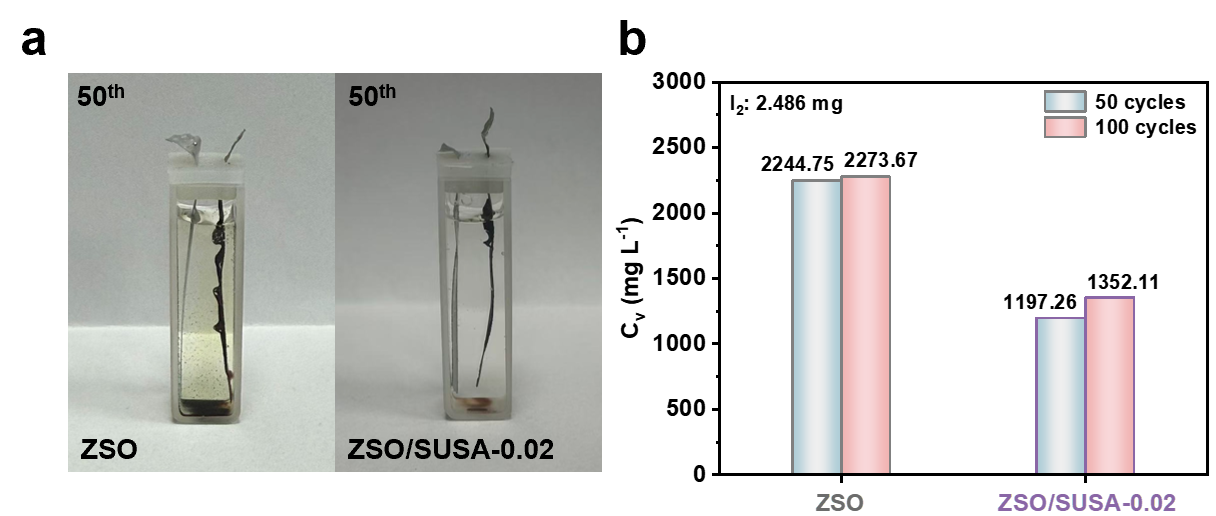


**Fig. S33 a** Electrolyte appearance post-50-cycling: (Left) yellowish ZSO electrolyte indicates I_2_ dissolution. (Right) clear ZSO/SUSA-0.02 electrolyte. **b** ICP-MS test of the electrolytes cycled in ZSO and ZSO/SUSA-0.02.

**Table S1** The comparative analysis of Zn||Cu cells performance in this work and other works

| **Current density**  **(mA cm^-2^)** | **Avg. CE (%)** | **Cycle number** | **Strategies** | **Ref.** |
| --- | --- | --- | --- | --- |
| 0.5 | 99.32 | 220 | Li_2_SO_4_ | 10.1021/acsami.2c18154 [S2] |
| 5 | 99.4 | 550 | ZG | 10.1016/j.mtener.2023.101279 [S3] |
| 2 | 99.35 | 800 | LA | 10.1002/adfm.202411477 [S4] |
| 5 | 99.71 | 400 | SN | 10.1002/adfm.202300795 [S5] |
| 1 | 97.8 | 300 | PV | 10.1002/adfm.202504195 [S6] |
| 0.5 | 99.16 | 550 | Rb_2_SO_4_ | 10.1002/smll.202303906 [S7] |
| 2 | 99.3 | 500 | Py | 10.1002/anie.202302302 [S8] |
| 1 | 99.24 | 800 | Caf | 10.1016/j.jechem.2025.02.066 [S9] |
| 5 | 99.6 | 600 | MPC | 10.1007/s40820-025-01826-w [S10] |
| 1 | 99.33 | 930 | SUSA | This work |
| 2 | 99.48 | 1600 | SUSA | This work |
| 5 | 99.76 | 1100 | SUSA | This work |

**Table S2** The comparative analysis of Zn||Zn cells performance (CPC) in this work and other works

| **Cycle time (h)** | **Current density (mA cm^-2^)** | **CPC (Ah cm^-2^)** | **Strategies** | **Ref.** |
| --- | --- | --- | --- | --- |
| 50 | 20 | 1 | SL | 10.1002/anie.202214966 [S11] |
| 275 | 5 | 1.375 | Glucose | 10.1002/anie.202105756 [S12] |
| 550 | 10 | 5.5 | Sac | 10.1002/adma.202100445 [S13] |
| 800 | 5 | 4 | Ser | 10.1002/aenm.202301517 [S14] |
| 530 | 10 | 5.3 | TOCN-CL | 10.1002/adfm.202422159 [S15] |
| 240 | 10 | 2.4 | OPDP | 10.1002/adfm.202509652 [S16] |
| 500 | 8 | 4 | HDO | 10.1002/anie.202518262 [S17] |
| 540 | 1 | 0.54 | NMP | 10.1002/aenm.202103231 [S18] |
| 700 | 5 | 3.5 | TH | 10.1016/j.nanoen.2022.107145 [S19] |
| 230 | 5 | 1.15 | MG | 10.1002/smll.202509633 [S20] |
| 346 | 8 | 2.768 | EZN | 10.1016/j.est.2025.118388 [S21] |
| 800 | 1 | 0.8 | CMCS | 10.1016/j.jcis.2025.138847 [S22] |
| 250 | 3 | 0.75 | CuGly | 10.1016/j.cej.2025.167617 [S23] |
| 320 | 10 | 3.2 | PD | 10.1039/d5ta04776e [S24] |
| 880 | 1 | 0.88 | Cre | 10.1002/smtd.202500188 [S25] |
| 675 | 10 | 6.75 | SUSA | This work |
| 1660 | 5 | 8.3 | SUSA | This work |

**Table S3** The comparative analysis of Zn||Zn cells performance (DOD) in this work and other works

| **Cycle time (h)** | **Current density**  **(mA cm^-2^)** | **DOD (%)** | **Strategies** | **Ref.** |
| --- | --- | --- | --- | --- |
| 160 | 10 | 6 | α-CD | 10.1021/jacs.2c00551 [S26] |
| 200 | 5 | 30 |  |  |
| 135 | 8 | 45.54 | DM | 10.1002/anie.202403050 [S27] |
| 240 | 5 | 42.7 | CI-GQDs | 10.1021/acsnano.3c04155 [S28] |
| 90 | 10 | 50 | Sr(NO_3_)_2_ | 10.1002/adfm.202401537 [S29] |
| 200 | 0.5 | 73.9 | NPA | 10.1016/j.ensm.2025.104727 [S30] |
| 200 | 2 | 34.3 | Zn@ZnS | 10.1007/s40820-023-01312-1 [S31] |
| 110 | 5 | 28 | CNT-Zn | 10.1002/adma.201903675 [S32] |
| 270 | 10 | 50 | ACE | 10.1016/j.cej.2023.146534 [S33] |
| 180 | 2 | 35 | EC | 10.1039/d3sc01831h [S34] |
| 400 | 3 | 51.24 | SUSA | This work |
| 350 | 10 | 56.9 | SUSA | This work |

**Supplementary References**

[S1] Y. Chen, S. Zhou, J. Li, X. Zhang, C. Zhou et al. Tuning Zn^2+^ deposition kinetics towards deep‐reversible zinc metal batteries with all‐climate adaptability. Angew. Chem. Int. Ed. **64**(18), e202423252 (2025). <https://doi.org/10.1002/anie.202423252>

[S2] H. K. Bezabh, J.-C. Chiou, T. A. Nigatu, T. M. Hagos, S.-K. Jiang et al. In-depth insight into a passive film through hydrogen-bonding network in an aqueous zinc battery. ACS Appl. Mater. Interfaces. **15**(6), 7949-7958 (2023). <https://doi.org/10.1021/acsami.2c18154>

[S3] R. Hao, S. Gu, Z. Wang, J. Chen, W. Luo et al. Reconstructing the solvation structure and solid-liquid interface enables dendrite-free zinc-ion batteries. Mater. Today Energy. **33**, 101279 (2023). <https://doi.org/10.1016/j.mtener.2023.101279>

[S4] R. Jiang, T. Naren, Y. Chen, Z. Chen, C. Zhang et al. Enhanced hydrogen bonding through strong water‐locking additives for long‐term cycling of zinc ion batteries. Adv. Funct. Mater. **34**(49), 2411477 (2024). <https://doi.org/10.1002/adfm.202411477>

[S5] N. Wang, X. Chen, H. Wan, B. Zhang, K. Guan et al. Zincophobic electrolyte achieves highly reversible zinc‐ion batteries. Adv. Funct. Mater. **33**(27), 2300795 (2023). <https://doi.org/10.1002/adfm.202300795>

[S6] W. Liang, D. Li, R. Zhong, S. Tao, Y. Zhu et al. Electrolyte engineering strategy with catecholate type additive enabled ultradurable Zn anode. Adv. Funct. Mater. **35**(36), 2504195 (2025). <https://doi.org/10.1002/adfm.202504195>

[S7] X. Zhang, J. Chen, H. Cao, X. Huang, Y. Liu et al. Efficient suppression of dendrites and side reactions by strong electrostatic shielding effect via the additive of Rb_2_SO_4_ for anodes in aqueous zinc‐ion batteries. Small. **19**(52), 2303906 (2023). <https://doi.org/10.1002/smll.202303906>

[S8] J. Luo, L. Xu, Y. Zhou, T. Yan, Y. Shao et al. Regulating the inner Helmholtz plane with a high donor additive for efficient anode reversibility in aqueous Zn‐ion batteries. Angew. Chem. Int. Ed. **62**(21), e202302302 (2023). <https://doi.org/10.1002/anie.202302302>

[S9] Z. Luo, T. Xu, L. Cao, Q. Liu, D. Li et al. Caffeine-enhanced Zn (002) texture-oriented growth for stable aqueous zinc-ion batteries. J. Energy Chem. **107**, 44-52 (2025). <https://doi.org/10.1016/j.jechem.2025.02.066>

[S10] B. Wang, C. Guan, Q. Zhou, Y. Wang, Y. Zhu et al. Screening anionic groups within zwitterionic additives for eliminating hydrogen evolution and dendrites in aqueous zinc ion batteries. Nano-Micro Lett. **17**(1), 314 (2025). <https://doi.org/10.1007/s40820-025-01826-w>

[S11] M. Wang, J. Ma, Y. Meng, J. Sun, Y. Yuan et al. High‐capacity zinc anode with 96 % utilization rate enabled by solvation structure design. Angew. Chem. Int. Ed. **62**(3), e202214966 (2022). <https://doi.org/10.1002/anie.202214966>

[S12] P. Sun, L. Ma, W. Zhou, M. Qiu, Z. Wang et al. Simultaneous regulation on solvation shell and electrode interface for dendrite‐free Zn ion batteries achieved by a low‐cost glucose additive. Angew. Chem. Int. Ed. **60**(33), 18247-18255 (2021). <https://doi.org/10.1002/anie.202105756>

[S13] C. Huang, X. Zhao, S. Liu, Y. Hao, Q. Tang et al. Stabilizing zinc anodes by regulating the electrical double layer with saccharin anions. Adv. Mater. **33**(38), 2100445 (2021). <https://doi.org/10.1002/adma.202100445>

[S14] Y. Wang, L. e. Mo, X. Zhang, Y. Ren, T. Wei et al. Facet‐termination promoted uniform Zn (100) deposition for high‐stable zinc‐ion batteries. Adv. Energy Mater. **13**(31), 2301517 (2023). <https://doi.org/10.1002/aenm.202301517>

[S15] Q. Ma, W. Song, X. Zhang, N. Yang, B. Wu et al. Reforming multifunctional solid electrolyte interphase for high‐performance Zn anode through a nature‐inspired strategy. Adv. Funct. Mater. **35**(19), 2422159 (2025). <https://doi.org/10.1002/adfm.202422159>

[S16] Y. Deng, Z. Xiao, W. Xu, Y. Wang, Z. Peng et al. Moderate yet reversible oligozwitterion adsorption unlocks stable zinc‐ion batteries. Adv. Funct. Mater. **35**(52), e09652 (2025). <https://doi.org/10.1002/adfm.202509652>

[S17] J. Wang, H. Yang, Y. Zhong, J. Feng, Z. Cui et al. A theory‐driven moderation strategy for electrolyte design unlocks stable aqueous zinc deposition. Angew. Chem. Int. Ed. **64**(51), e202518262 (2025). <https://doi.org/10.1002/anie.202518262>

[S18] T. C. Li, Y. V. Lim, X. L. Li, S. Luo, C. Lin et al. A universal additive strategy to reshape electrolyte solvation structure toward reversible Zn storage. Adv. Energy Mater. **12**(15), 2103231 (2022). <https://doi.org/10.1002/aenm.202103231>

[S19] Z. Miao, Q. Liu, W. Wei, X. Zhao, M. Du et al. Unveiling unique steric effect of threonine additive for highly reversible Zn anode. Nano Energy. **97**, 107145 (2022). <https://doi.org/10.1016/j.nanoen.2022.107145>

[S20] Z. Wang, R. Cao, J. Lu, R. Yang, L. Qin et al. Natural mogroside additive enabling stable zinc anodes through solvation and hydrogen bond network regulation. Small. **21**(49), e09633 (2025). <https://doi.org/10.1002/smll.202509633>

[S21] L. Shang, C. Cao, J. Fan, L. Yuan. Ethylenediaminetetraacetic acid zinc disodium salt electrolyte additive for enhanced zinc anode performance in aqueous zinc-ion batteries. J. Energy Storage. **136**, 118388 (2025). <https://doi.org/10.1016/j.est.2025.118388>

[S22] K. Zhao, S. Ma, J. Zhao, H. Li, C. Ma et al. Synergistic interfacial chemistry enabled by a multifunctional zwitterionic polymer additive for highly reversible Zn metal anodes. J. Colloid Interface Sci. **702**, 138847 (2026). <https://doi.org/10.1016/j.jcis.2025.138847>

[S23] L. Chen, Y. Tian, B.-C. Liu, B.-X. Zhang, K.-R. Ren et al. Stabilizing zinc anode with multifunctional glycine copper additive. Chem. Eng. J. **522**, 167617 (2025). <https://doi.org/10.1016/j.cej.2025.167617>

[S24] J. Yang, T. Sun, M. Cheng, W. Zhang, Z. Zha et al. Adsorption-induced dual-layer solid electrolyte interface toward a highly reversible Zn anode. J. Mater. Chem. A. **13**(36), 30118-30127 (2025). <https://doi.org/10.1039/d5ta04776e>

[S25] Y. Lu, Y. Jiang, J. Yi, Y. Wei, F. Wang et al. Creatinine: A muscle metabolite as a multifunctional electrolyte additive for aqueous zinc‐ion batteries. Small Methods. **9**(10), 2500188 (2025). <https://doi.org/10.1002/smtd.202500188>

[S26] K. Zhao, G. Fan, J. Liu, F. Liu, J. Li et al. Boosting the kinetics and stability of Zn anodes in aqueous electrolytes with supramolecular cyclodextrin additives. J. Am. Chem. Soc. **144**(25), 11129-11137 (2022). <https://doi.org/10.1021/jacs.2c00551>

[S27] S. Zhou, X. Meng, Y. Chen, J. Li, S. Lin et al. Zinc‐ion anchor induced highly reversible Zn anodes for high performance Zn‐ion batteries. Angew. Chem. Int. Ed. **63**(24), e202403050 (2024). <https://doi.org/10.1002/anie.202403050>

[S28] H. Wang, A. Zhou, X. Hu, Z. Hu, F. Zhang et al. Bifunctional dynamic adaptive interphase reconfiguration for zinc deposition modulation and side reaction suppression in aqueous zinc ion batteries. ACS Nano. **17**(12), 11946-11956 (2023). <https://doi.org/10.1021/acsnano.3c04155>

[S29] J. Cao, J. Wu, H. Wu, Y. Jin, D. Luo et al. Dendrite‐free zinc anode via oriented plating with alkaline earth metal ion additives. Adv. Funct. Mater. **34**(32), 2401537 (2024). <https://doi.org/10.1002/adfm.202401537>

[S30] Z. Liu, J. Sun, X. Li, N. Jiang, Y. Zhang et al. Constructing eutectic solvation sheath by weak solvation effect for stabilizing Zn-ion batteries with low-temperature adaptability. Energy Storage Mater. **83**, 104727 (2025). <https://doi.org/10.1016/j.ensm.2025.104727>

[S31] Y. Chen, Z. Deng, Y. Sun, Y. Li, H. Zhang et al. Ultrathin zincophilic interphase regulated electric double layer enabling highly stable aqueous zinc-ion batteries. Nano-Micro Lett. **16**(1), 96 (2024). <https://doi.org/10.1007/s40820-023-01312-1>

[S32] Y. Zeng, X. Zhang, R. Qin, X. Liu, P. Fang et al. Dendrite‐free zinc deposition induced by multifunctional cnt frameworks for stable flexible Zn‐ion batteries. Adv. Mater. **31**(36), 1903675 (2019). <https://doi.org/10.1002/adma.201903675>

[S33] Q. Guan, J. Li, L. Li, P. Chai, Y. Li et al. In situ construction of organic anion-enriched interface achieves ultra-long life aqueous zinc-ion battery. Chem. Eng. J. **476**, 146534 (2023). <https://doi.org/10.1016/j.cej.2023.146534>

[S34] K. Wang, T. Qiu, L. Lin, F. Liu, J. Zhu et al. Interface solvation regulation stabilizing the Zn metal anode in aqueous Zn batteries. Chem. Sci. **14**(30), 8076-8083 (2023). <https://doi.org/10.1039/d3sc01831h>
